# Analysis of Alkylresorcinol, Phytosterol, Carotenoid, and Vitamin E Contents in Korean Wheat Cultivars

**DOI:** 10.3390/foods15061075

**Published:** 2026-03-19

**Authors:** Huijin Heo, Seonghwa Hong, Jinhee Park, Kyeong-Hoon Kim, Heon-Sang Jeong, Hana Lee, Junsoo Lee

**Affiliations:** 1 Department of Food Science and Biotechnology, Chungbuk National University, Cheongju 28644, Chungbuk, Republic of Korea; pltreasure11@gmail.com (H.H.); tjd3465@naver.com (S.H.); hsjeong@chungbuk.ac.kr (H.-S.J.); 2 Wheat Research Team, National Institute of Crop Science, Wanju 55365, Jeonbuk, Republic of Korea; pjh237@korea.kr (J.P.); k2h0331@korea.kr (K.-H.K.)

**Keywords:** phytochemicals, whole wheat, cultivars, year variation

## Abstract

This study investigated the phytochemical profiles of 41 Korean wheat cultivars harvested over two consecutive years (2019 and 2020), with a focus on alkylresorcinols (ARs), phytosterols, vitamin E, and carotenoids. Validated chromatographic analyses revealed considerable variation among cultivars. AR levels, particularly heneicosylresorcinol, showed relatively consistent patterns across years, whereas the concentrations of phytosterols, vitamin E, and carotenoids varied more noticeably between years, suggesting possible associations with environmental conditions. Hierarchical clustering analysis classified the cultivars into five distinct groups according to their overall phytochemical profiles. ‘Dajoong’ and ‘Shinmichal’ exhibited the highest AR levels; ‘Hanbaek’, ‘Goso’, and ‘Joah’ were richest in β-sitosterol; ‘Eunpa’ and ‘Namhae’ showed elevated β-tocotrienol content, while ‘Uri’ and ‘Chungkye’ were notable for high lutein concentrations. ‘Saekeumkang’ displayed a balanced profile across all phytochemical classes. These findings provide baseline data on phytochemical variation among Korean wheat cultivars and offer insight into differences in phytochemical diversity.

## 1. Introduction

Wheat (*Triticum aestivum* L.) is one of the most extensively produced crops globally, with an annual production exceeding 780 million tons worldwide in recent years, according to FAO statistics [1]. It has long been recognized as an essential food source, providing not only carbohydrates but also various bioactive compounds beneficial for human health [2]. Whole wheat grains, including the bran and germ, are particularly rich in health-promoting phytochemicals such as phenolic acids, flavonoids, tocols, alkylresorcinols (ARs), and phytosterols [3]. Consuming whole wheat is associated with health benefits, including improved digestive health and reduced risks of chronic diseases, such as heart disease, obesity, and type 2 diabetes [4,5]. With the growing interest in functional foods and plant-based diets, research has increasingly focused on understanding the nutritional value of whole grains.

Whole wheat is a rich source of phytochemicals including ARs, phytosterols, vitamin E, and carotenoids, which contribute to its antioxidant, anti-inflammatory, and protective effects against chronic diseases [6]. ARs, also known as resorcinol lipids, are phenolic lipids characterized by odd-numbered saturated aliphatic side chains and resorcinol-type phenolic rings. ARs typically feature a saturated aliphatic side chain containing 17, 19, 21, 23, or 25 carbon atoms [3,7]. ARs exhibit various bioactive properties, including antimicrobial activity, neuroprotection, anti-aging effects, and wound healing. They have shown promise in preventing muscle atrophy, improving metabolic health, and reducing cancer risk, making ARs potentially valuable for health-related applications [8]. Phytosterols, such as campesterol, stigmasterol, and β-sitosterol, are found in the germ and bran layers of wheat kernels and are known to interfere with dietary and biliary cholesterol absorption in the small intestine [9]. Whole wheat is a rich source of vitamin E, which includes four tocopherols (α-, β-, γ-, and δ-T) and the corresponding tocotrienols (α-, β-, γ-, and δ-T3), all of which provide antioxidant benefits [10]. Carotenoids are natural pigments in wheat that contribute to its golden color and protect cells from oxidative damage [11]. These compounds provide primary protection and exhibit synergistic effects in cell membranes.

Given the broad range of physiological benefits associated with these compounds, interest in strategies to enhance phytochemical intake through commonly consumed foods has grown. These efforts include the development and consumption of whole-grain products designed to deliver health-promoting compounds through staple foods such as bread, pasta, and breakfast cereals. However, the concentration and distribution of these compounds vary significantly across different wheat cultivars and are influenced by various factors, such as genotype, growing conditions, and environmental stressors [12]. A previous study reported that the total AR content in 175 wheat genotypes varied between 19.4 and 74.1 mg/100 g dry matter [13]. Phytosterol contents of 195 wheat cultivars ranged from 6.7−11.87 mg/100 g dry weight [14]. The primary carotenoid (lutein) was present in the eight wheat cultivars at levels ranging from 82–114 µg/100 g dry weight, whereas α-T was found in all wheat grains, ranging from 0.34–1.01 mg/100 g dry weight [15]. Previous studies on Korean wheat cultivars have primarily focused on the general chemical composition of whole grain wheat flour, including ash, lipid, protein, dietary fiber, and carbohydrate contents, and have reported substantial variation among genotypes [16]. These studies collectively demonstrate that the concentrations of major phytochemicals in wheat can vary widely among cultivars depending on their genetic background. Although considerable variability in phytochemical composition has been reported among wheat genotypes, systematic multi-year assessments of these constituents in Korean wheat cultivars remain limited. Therefore, the primary objective of this study was to systematically evaluate and compare the contents of ARs, phytosterols, vitamin E, and carotenoids in the whole grains of 41 major Korean wheat cultivars. By analyzing samples harvested over two consecutive years (2019 and 2020), this study also aimed to explore potential year-to-year variation in phytochemical concentrations, providing preliminary insight into the possible influence of environmental conditions on phytochemical profiles.

## 2. Materials and Methods

### 2.1. Materials

This study investigated 41 Korean cultivars, which were provided by the Rural Development Administration (RDA) of the Republic of Korea and cultivated in 2019 and 2020. The 41 cultivars were included as part of a domestic research project evaluating currently cultivated wheat varieties in Korea. Cultivars were grown at the upland crop experimental farm of the National Institute of Crop Science, RDA, on the same field plots each year. Fertilizer was applied at a ratio of 9.1:7.4:3.9 kg/10 acres (N:P:K) before sowing. During the growth period, rigorous pest and disease management was implemented, and no supplemental irrigation was applied. Sowing occurred in October, and harvesting was completed between 5–10 June of each subsequent year. As the grains were harvested at full maturity, their final moisture content was approximately 12–14%. The experiment followed a randomized complete block design with three replicates per cultivar and year. After harvesting, the grains from each replicate plot were separately dried using forced-air dryers. To ensure consistency in quality assessment and minimize within-replicate variability, the dried grains obtained from three biological replicates were pooled to form a single composite sample for each cultivar in a given year. Compositional analyses were performed in duplicate for each composite sample. Information on the wheat cultivars assessed in this study is provided in Table 1 and Table S1.

### 2.2. Pretreatment Carotenoid, Vitamin E, and Phytosterol Analysis

Whole wheat samples (100 g) from 41 wheat cultivars were milled using a Wiswell grinder (Model SP-7426; Supreme Electric Manufacture Co., Ltd., Guangzhou, China) and passed through a 30-mesh sieve to ensure uniform particle size prior to extraction. Saponification was employed as a pretreatment method for the analysis of phytosterols, vitamin E, and carotenoids [17]. Specifically, 2 g whole wheat powder was saponified with 20 mL of a 6% pyrogallol solution in ethanol, followed by the addition of 8 mL of a 60% (*w*/*v*) potassium hydroxide solution. The mixture was heated to reflux at 75 ± 1 °C for 50 min with agitation in a water bath (NTS-1300; Eyela, Tokyo, Japan). After heating, the mixture was rapidly cooled in an ice bath. Next, 30 mL of a 2% sodium chloride solution and 20 mL of n-hexane containing 0.01% butylated hydroxytoluene were added for extraction. The mixture was vortexed for 2 min and allowed to phase-separate, after which the hexane layer was collected in a 50 mL volumetric flask. The hexane extract, which contained the targeted phytochemicals, was filtered through a 0.45 µm Polytetrafluoroethylene (PTFE) membrane filter before injection into the high-performance liquid chromatography (HPLC) system for analysis. For the measurement of phytosterols, the hexane extract was evaporated under nitrogen, and trimethylsilyl derivatives of the phytosterols were prepared by adding 100 μL of pyridine and N-methyl-N-trimethylsilylfluoroacetamide. The tubes were shaken to dissolve the sample in the reagent and then heated at 60 °C for 20 min. Excess reagent was subsequently removed under nitrogen. One milliliter of dimethylformamide was added to the remaining residue, and the sample was filtered through a 0.45 μm PTFE filter and injected into the gas chromatography (GC) system for analysis.

### 2.3. Pretreatment for the Analysis of Alkylresorcinols

Each ground whole wheat grain sample (1.0 g) was spiked with methyl behenate (0.5 mg/mL) as the internal standard (ISTD) for GC quantification. Whole wheat powder was extracted with 40 mL ethyl acetate at room temperature for a 24-h period using a shaker. The organic phases were then collected and the volume adjusted to 50 mL, resulting in a solution containing ARs. For analysis, 4 mL extract solvent was evaporated under a nitrogen stream. The ARs were derivatized into their trimethylsilyl ether forms via addition of 100 µL BSTFA + TMCS + TMSI silylating reagent. The samples were dissolved through shaking and heating at 65 °C for 30 min. Excess reagent was removed under nitrogen and the residue redissolved in ethyl acetate. After filtration through a 0.45 µm PTFE membrane filter, the samples were ready for injection into the GC system. All solvents used throughout the process were of HPLC grade.

### 2.4. High-Performance Liquid Chromatography Conditions for Carotenoid Analysis

Carotenoids were analyzed using an HPLC system equipped with a UV-2075 UV detector, PU-2089 pump, AS-2055 auto-injector, and CO-2060 column oven (JASCO Corp., Tokyo, Japan), along with a Vydac 201TP54 column (250 × 4.6 mm, 5 μm; Grace, Columbia, MD, USA). An isocratic elution was employed using acetonitrile:methanol:1,2-dichloroethane (65:30:5, *v*/*v*) containing 0.05 M ammonium acetate in methanol as the mobile phase. The flow rate was set at 1.0 mL/min, and the column temperature was maintained at 25 °C. Detection was performed at 450 nm. Quantification was performed via external calibration using lutein and zeaxanthin, with calibration curve equations provided in Table S2. Carotenoid peaks were identified by comparing retention times with those of authentic standards, as shown in the HPLC chromatograms of whole wheat samples (Figure S1).

### 2.5. High-Performance Liquid Chromatography Conditions for Vitamin E Analysis

Based on the method reported by Yu et al. (2023) [18], tocopherols and tocotrienols were analyzed using an HPLC system equipped with an FP-2020 fluorescence detector, PU-2089 pump, AS-2055 auto-injector, and CO-2060 column oven (JASCO Corp.), as well as a LiChrosphere Diol 100 HPLC column (250 × 4 mm, i.d., 5 μm; Merck, Darmstadt, Germany). An isocratic elution was employed using hexane:isopropanol (98.7:1.3, *v*/*v*) as the mobile phase, at a flow rate of 1.0 mL/min. The column temperature was maintained at 25 °C. Detection was performed at excitation and emission wavelengths of 290 and 330 nm, respectively. Quantification was achieved using external calibration with α-T, β-T, α-T3, and β-T3, and the calibration curve equations are provided in Table S2. Vitamin E peaks were identified by comparing retention times with those of authentic standards, as shown in the HPLC chromatogram (Figure S2).

### 2.6. Gas Chromatography Conditions for Phytosterol Analysis

For the determination of phytosterols, all samples were analyzed via GC (7890A; Agilent, Santa Clara, CA, USA) using a SAC-5 Fused Silica Capillary column (30 m × 0.25 mm × 0.25 μm; Supelco, Bellefonte, PA, USA). A flame ionization detector was operated at 285 °C, and the injector was set at 300 °C. The column oven temperature was held at 285 °C for 20 min. High-purity (99.999%) N2 was used as a carrier gas at a flow rate of 1.0 mL/min. The injection volume was 2 µL with a split ratio of 10:1. Quantification was performed using 5α-cholestane as an ISTD and relative response factor (RRF), and concentration was calculated using the following formula:

Concentration = (Area of sample ÷ Area of ISTD) × Concentration of ISTD × RRF × Dilution factor.

Phytosterol peaks were identified by comparing retention times with those of authentic standards, as shown in the GC chromatogram (Figure S3).

### 2.7. Gas Chromatography Conditions for Alkylresorcinol Analysis

The conditions for AR analysis were based on the method described by Bordiga et al. (2016) [19], with slight modifications. For the determination of ARs, all samples were analyzed via GC using a capillary column (DB-17HT, 30 m × 0.25 mm × 0.1 μm; Agilent). A flame ionization detector was set to 350 °C, and the injector set at 250 °C. The column oven temperature was held at 150 °C for 2 min, then programmed to increase to 320 °C at a rate of 10 °C/min and maintained for 7 min. High-purity (99.999%) N_2_ was used as a carrier gas at a flow rate of 1.0 mL/min. The injection volume was 2 µL with a split ratio of 10:1. Quantification was performed using methyl behenate as an ISTD and RRF, and concentrations were calculated using the formula shown in Section 2.6. AR peaks were identified by comparing retention times with those of authentic standards, as shown in the GC chromatogram (Figure S4).

### 2.8. Method Validation

All analytical methods used in this study were validated by assessing their precision (repeatability and reproducibility) and accuracy (recovery). Repeatability was evaluated through five independent analyses of replicate samples conducted on the same day, whereas reproducibility was determined through five separate analyses of replicate samples performed on different days. Precision results are expressed as mean, standard deviation (SD), and coefficient of variation (CV). Accuracy was assessed using recovery experiments performed with representative compounds for each phytochemical class: heptadecylresorcinol for alkylresorcinols, β-sitosterol for phytosterols, α-tocopherol for vitamin E, and lutein for carotenoids. Recovery (%) was calculated using the following equation: recovery (%) = (Cs − Cp)/Ca × 100, where Cs is the amount of target compound present in the spiked sample, Cp is the amount of target compound present in the sample, and Ca is the amount of target compound added. Calibration curves, coefficients of determination (R^2^), limits of detection (LOD), and limits of quantification (LOQ) were determined for compounds analyzed by HPLC using external standards, whereas alkylresorcinols and phytosterols analyzed by GC were quantified using an ISTD and RRF.

### 2.9. Statistical Analysis

Box–whisker plots were constructed using GraphPad Prism (version 8.2.1; GraphPad Software, San Diego, CA, USA). A box-and-whisker plot consists of a rectangular box representing the middle 50% of the data (interquartile range), with the line inside representing the median. The whiskers extend from the box to show minimum and maximum values, representing the spread of the data. Hierarchical clustering analysis was performed using the Euclidean distance metric and Ward’s linkage method (Ward.D2) in RStudio (version 4.2.1), and the results were visualized as a heatmap. Prior to clustering analysis, phytochemical variables were standardized using z-score scaling to remove scale differences among variables. The dendrogram was visually inspected, and the cultivars were grouped into five clusters (A–E) at a level that provided clear separation of phytochemical composition patterns while maintaining interpretable cluster sizes. Principal component analysis (PCA) was performed using RStudio with the Factoextra package (fviz_pca_biplot function) to visualize overall variation in phytochemical composition among the 41 wheat cultivars. Thirteen phytochemical compounds, including alkylresorcinols (C17:0, C19:0, C21:0, C23:0), phytosterols (campesterol, stigmasterol, β-sitosterol), vitamin E homologues (α-T, α-T3, β-T, β-T3), and carotenoids (lutein, zeaxanthin) were used as input variables. Patterns in PCA were interpreted based on the separation of cultivars along the first two principal components, which captured the largest proportions of total variation, allowing identification of cultivars with similar or divergent phytochemical profiles. Differences in phytochemical contents among cultivars were evaluated using one-way analysis of variance (ANOVA) followed by Tukey’s honestly significant difference (HSD) test (*p* < 0.05). In addition, two-way ANOVA was performed using GraphPad Prism to evaluate the effects of cultivar and harvest year on phytochemical levels, with cultivar and year treated as fixed factors. Differences in phytochemical contents between harvest years within the same cluster group were evaluated using a two-tailed *t*-test (*p* < 0.05). The distribution characteristics of the phytochemical variables, including skewness and kurtosis, are summarized in Table 2. Skewness ranged from –0.73 to 1.30 and kurtosis from –1.34 to 3.12, which fall within commonly accepted ranges for approximate normality (skewness < |2|, kurtosis < |7|). These results indicate that the data did not substantially deviate from normality. In addition, group sample sizes were balanced, under which ANOVA is generally robust to moderate departures from normality and homogeneity of variance. Each phytochemical compound was treated as an independent response variable and analyzed separately; therefore, correction for multiple testing across different phytochemicals was not applied.

## 3. Results and Discussions

### 3.1. Variability of Phytochemical Content in Whole Wheat Based on Descriptive Statistics

The contents of ARs, phytosterols, vitamin E, and carotenoids in 41 wheat cultivars across two harvest years are presented in the Supplementary Data (Tables S3–S6). Descriptive statistics for each compound—including minimum, maximum, mean, standard deviation (SD), coefficient of variation (CV), skewness, and kurtosis—are presented in Table 2 [20]. In the present study, heneicosylresorcinol (C21:0) had a mean content of 25.81 mg/100 g in 2019 and 24.91 mg/100 g in 2020, showing the highest level among the ARs regardless of the harvest year, followed by nonadecylresorcinol (C19:0), tricosylresorcinol (C23:0), and heptadecylresorcinol (C17:0). Among the ARs, heneicosylresorcinol (C21:0) had the highest content and lowest variability (CVs of 15.86% and 13.45% in 2019 and 2020, respectively), whereas heptadecylresorcinol (C17:0) had the lowest content and highest variability (CVs of 22.26% and 21.73%). These results are generally similar to those reported in previous studies for wheat phytochemicals. For example, total AR content in wheat has been reported to vary between 19.4 and 74.1 mg/100 g dry matter, phytosterol contents ranged from 6.7 to 11.87 mg/100 g dry weight, lutein levels ranged from 82–114 µg/100 g dry weight, and α-tocopherol (α-T) ranged from 0.34–1.01 mg/100 g dry weight [13,14,15]. These comparisons indicate that the observed phytochemical concentrations in the studied cultivars are generally consistent with earlier findings, while higher values in certain domestic cultivars likely reflect genetic and environmental influences.

β-Sitosterol was the predominant phytosterol, accounting for approximately 40–60% of the total phytosterol composition in whole wheat [14]. The kurtosis and CV values of stigmasterol were higher than those of the other phytosterols, indicating that the stigmasterol content varied considerably among the different wheat cultivars. Several vitamin E forms (γ-T, γ-T3, δ-T, and δ-T3) were detected only at trace levels in the chromatograms and remained below the quantification limits of the analytical method (Table S2); therefore, they were not included in the quantitative analysis. The absence of quantifiable γ- and δ-forms of vitamin E in our whole wheat samples is consistent with prior findings that these forms are typically undetectable or present only in trace amounts in wheat. Previous studies have consistently shown that α- and β-tocopherols and tocotrienols are the predominant tocochromanols in wheat grain, while γ- and δ-isomers are either absent or present at very low levels [21,22,23,24]. Among the detected vitamin E forms, α-T3 in 2019 and β-T3 in 2020 exhibited the highest CV variability at 38.75% and 40.51%, respectively. This indicates that a significant variation in vitamin E content occurred depending on the harvest year. The zeaxanthin content was lower than that of lutein in all wheat cultivars. The average lutein content in harvested whole wheat was 93.24 µg/100 g in 2019 and 67.30 µg/100 g in 2020. Although the zeaxanthin content was consistently lower than that of lutein, its variability across wheat cultivars was greater, as evidenced by a larger CV measured for it than for lutein. These findings highlight the considerable variability in phytochemical content among wheat cultivars, emphasizing the influence that genetic factors exert.

### 3.2. Impact of Environmental Factors on Annual Variation in Phytochemical Content

**Figure 1:**
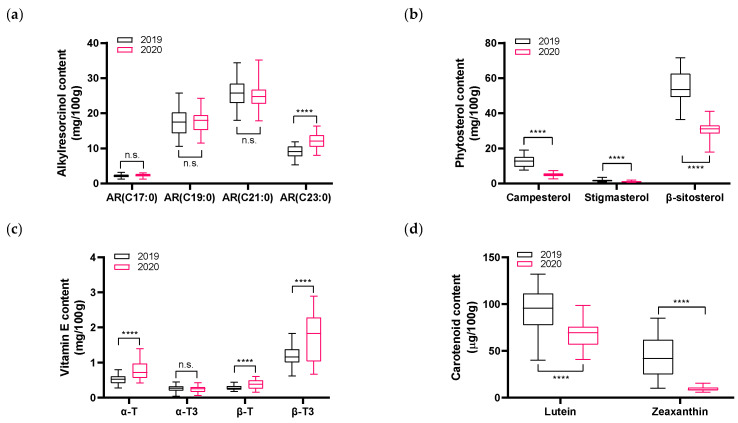
Effect of harvest year on phytochemical concentrations in wheat cultivars grown in two consecutive years (2019 and 2020). Box-whisker plots illustrate the content of (**a**) alkylresorcinols (C17:0, heptadecylresorcinol; C19:0, nonadecylresorcinol; C21:0, heneicosylresorcinol; C23:0, tricosylresorcinol), (**b**) phytosterols (campesterol, stigmasterol, and β-sitosterol), (**c**) vitamin E (α-T, α-tocopherol; α-T3, α-tocotrienol; β-T, β-tocopherol; β-T3, β-tocotrienol), and (**d**) carotenoids (lutein and zeaxanthin). Statistical significance was assessed by two-way ANOVA with cultivar and year as factors. Asterisks indicate significant differences with **** *p* < 0.0001; n.s., not significant.

Annual variations in the phytochemical contents of whole wheat cultivars between 2019 and 2020 were evaluated using box-and-whisker plots (Figure 1). ARs exhibited minimal interannual variability, with stable levels across both years, suggesting a predominant influence of genetic factors. Among them, tricosylresorcinol (C23:0) showed slight year-to-year changes, but overall consistency was maintained (Figure 1a). Significant reductions were observed in phytosterols and carotenoids in 2020 relative to 2019 (Figure 1b,d). Vitamin E content showed moderate variation, with a broader range across cultivars (Figure 1c), indicating a more complex response involving both genetic and environmental factors. The reduction in phytosterols and carotenoids among the whole wheat varieties harvested in 2020 may be associated with differences in environmental conditions between the two growing seasons. In 2020, total sunlight exposure was reduced by approximately 119 h compared to 2019, while total precipitation increased by 156 mm (Table S7). Previous studies reported that light is crucial for the biosynthesis and accumulation of secondary metabolites, including phytosterols and carotenoids [25,26,27]. Additionally, total rainfall in 2020 increased by approximately 156 mm compared with that in 2019, with notable increases in precipitation occurring from 21 February–20 March and 21 May–20 June. Water is essential for plant survival; however, excessive amounts can cause stress and hinder the exchange of gases between the soil and atmosphere [28]. Moreover, excessive rainfall may lead to stomatal closure and decreased photosynthetic activity [29]. Reduced photosynthesis may influence secondary metabolite biosynthesis. These findings support the interpretation that AR levels are primarily genotype-driven, while phytosterol, vitamin E, and carotenoid concentrations are more environmentally responsive. Furthermore, field research on vegetables such as carrots, potatoes, and cabbage reported significant variation in phenolic compounds and antioxidant activity between years with different weather conditions (temperature, rainfall), indicating that seasonal environmental variability can contribute to phytochemical changes [30,31]. However, as the present study was conducted over only two growing seasons, the influence of environmental conditions on phytochemical accumulation could not be fully resolved. Further multi-year studies incorporating detailed meteorological data and physiological measurements are required to more clearly characterize these relationships in wheat.

To assess the relative influence of genetic and environmental factors at the composite sample level, a standard two-way ANOVA was performed for each phytochemical component, with cultivar and harvest year treated as fixed factors and their interaction included (Table S8). The F-statistics and corresponding *p*-values for each factor were used to evaluate the relative influence of cultivar and year. For alkylresorcinols, variation was largely associated with cultivar (C17:0: F_cultivar = 26.88, *p* < 0.0001; C19:0: F_cultivar = 58.49, *p* < 0.0001; C21:0: F_cultivar = 34.15, *p* < 0.0001), whereas the effect of year was minimal (C17:0: F_year = 13.45, *p* = 0.0004). In contrast, phytosterols such as campesterol and β-sitosterol were predominantly associated with year (campesterol: F_year = 2348, *p* < 0.0001; β-sitosterol: F_year = 3030, *p* < 0.0001). For vitamin E compounds, contributions from cultivar and year were more balanced (α-tocopherol: F_cultivar = 31.48, *p* < 0.0001; F_year = 920.0, *p* < 0.0001). For carotenoids, lutein and zeaxanthin were more strongly associated with year than cultivar (lutein: F_year = 1400, *p* < 0.0001; zeaxanthin: F_year = 12,818, *p* < 0.0001). Overall, these associations at the composite sample level suggest that AR levels are largely linked with genotype, whereas phytosterols and carotenoids show stronger associations with environmental conditions. This pattern aligns with previous studies reporting that genotype is the primary determinant of AR content in whole wheat [7,32], although environmental and meteorological factors may also contribute to variation [33,34]. Overall, our findings suggest that both genetic and environmental factors are associated with the phytochemical profiles of whole wheat.

From a nutritional and functional standpoint, such variability may influence the consistency and efficacy of health benefits associated with wheat-based foods. Since phytochemicals such as ARs, phytosterols, vitamin E, and carotenoids are known to contribute to antioxidant, anti-inflammatory, and cholesterol-lowering effects, differences in their levels could influence the nutritional quality of wheat-based foods [3]. Therefore, a comprehensive understanding of both genetic and environmental influences is essential for the selection of cultivars in breeding programs aimed at enhancing the nutritional quality of whole wheat.

### 3.3. Clustering Analysis of Phytochemical Profiles in 41 Wheat Cultivars Harvested in 2019 and 2020

**Figure 2:**
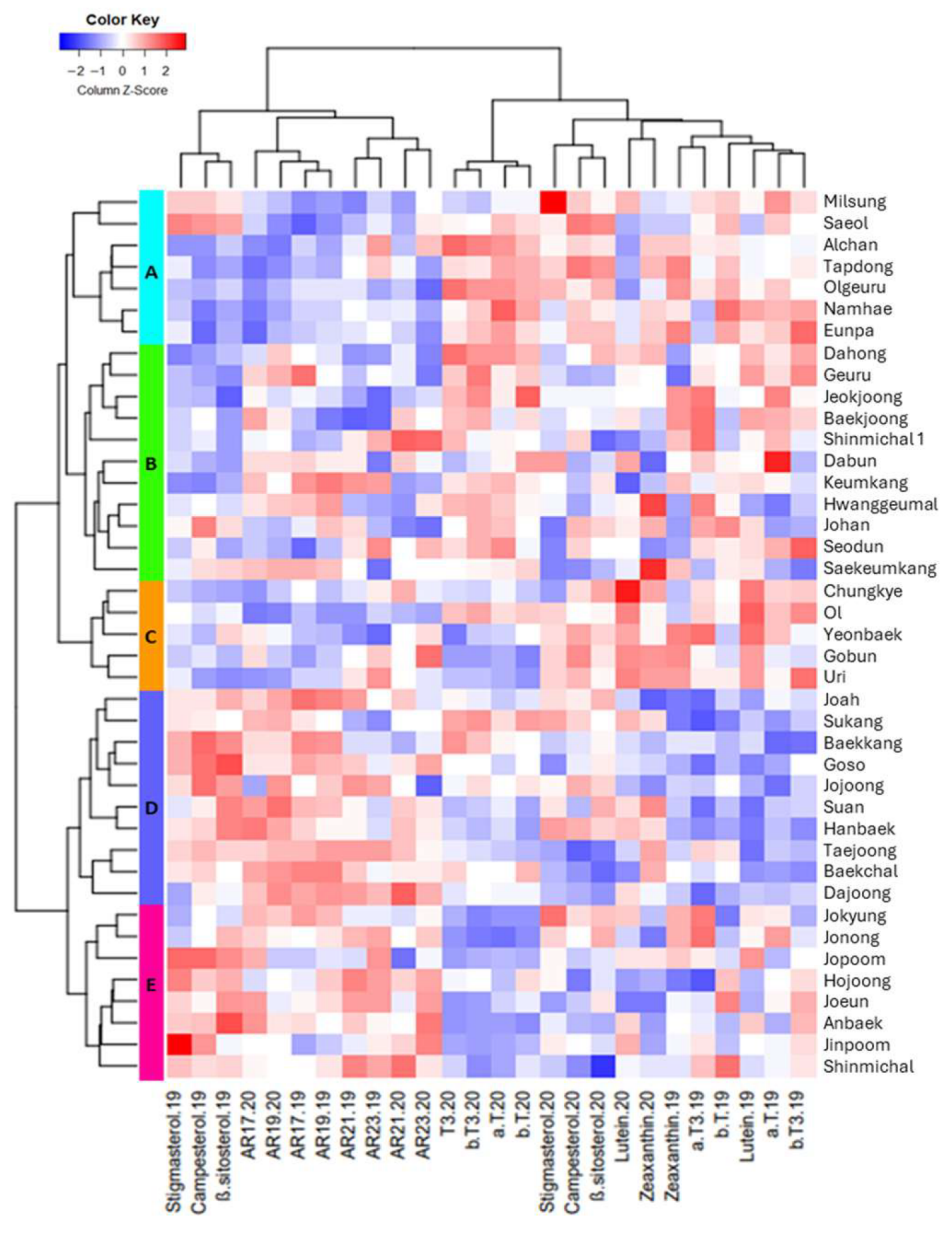
Hierarchical clustering of phytochemical profiles in 41 wheat cultivars. A heatmap illustrates the relative abundance of thirteen phytochemical compounds, including alkylresorcinols, phytosterols, vitamin E homologues, and carotenoids, across all cultivars. Each row represents an individual wheat cultivar, and each column corresponds to a specific phytochemical compound. The cultivars were clustered based on their overall phytochemical profiles, resulting in five distinct groups (A–E). The color intensity of each cell indicates the relative abundance of a particular phytochemical in a given cultivar, with red representing higher levels and blue representing lower levels.

Clustering analysis was conducted based on the contents of ARs, phytosterols, vitamin E, and carotenoids in 41 wheat cultivars harvested in 2019 and 2020. The cultivars were grouped into five clusters—A (7 cultivars), B (11), C (5), D (10), and E (8)—and group-wise averages were used to compare phytochemical profiles across years (Figure 2 and Table 3). Groups D and E exhibited the highest total AR contents, ranging from 45.39 to 59.94 mg/100 g in 2019, consistent across both years. The predominant homologs were C19:0 and C21:0, while C17:0 and C23:0 were present in lower concentrations, consistent with earlier reports [13]. These values fall within the previously reported range of 22.0–94.3 mg/100 g in wheat, although discrepancies among studies may be attributed to environmental influences and genetic variability [7]. Cultivars ‘Dajoong’ (Group D) and ‘Shinmichal’ (Group E) consistently exhibited the highest AR concentrations within their respective groups across both years. In 2019, Groups D and E exhibited the highest total phytosterol concentrations among all groups, particularly β-sitosterol, which was measured at 61.30 and 61.03 mg/100 g, respectively. Notably, cultivars ‘Hanbaek’, ‘Goso’, and ‘Joah’ (Group D), as well as ‘Anbaek’ and ‘Jonong’ (Group E), consistently exhibited high phytosterol content. The phytosterol levels observed in this study exceed previously reported ranges of 20.2 to 35.5 mg/100 g in whole wheat grain, where β-sitosterol, campesterol, and stigmasterol were identified as predominant components [35]. These findings indicate that certain domestic wheat cultivars may represent valuable sources of phytosterols, with potential for improved nutritional and functional properties.

Group A was characterized by the highest levels of α-tocopherol, β-tocopherol, and β-tocotrienol in both years. Within this group, cultivars ‘Eunpa’ and ‘Namhae’ showed the greatest vitamin E concentrations. Across all samples, β-tocotrienol was the dominant homolog, accounting for 50–55% of total vitamin E, in line with previous findings that reported β-T3 as the major form in wheat [24].

Carotenoid concentrations varied widely, with lutein and zeaxanthin levels ranging from 61.73–120.13 μg/100 g and 7.76–62.02 μg/100 g, respectively. Group C displayed the highest average lutein levels across both years, particularly cultivars ‘Uri’ and ‘Chungkye’. β-Carotene and α-carotene were not detected, consistent with prior reports [36], although other studies have reported detectable β-carotene and higher lutein values [24]. Such discrepancies likely reflect genetic differences among cultivars.

Interestingly, cultivar ‘Saekeumkang’ (Group B) exhibited a balanced phytochemical profile, with above-average levels across all four compound classes. This compositional balance may underlie its previously reported health benefits, including efficacy against non-alcoholic fatty liver disease and obesity [4,37], emphasizing the potential significance of phytochemical synergy in wheat cultivars.

### 3.4. Principal Component Analysis of Phytochemical Profiles

**Figure 3:**
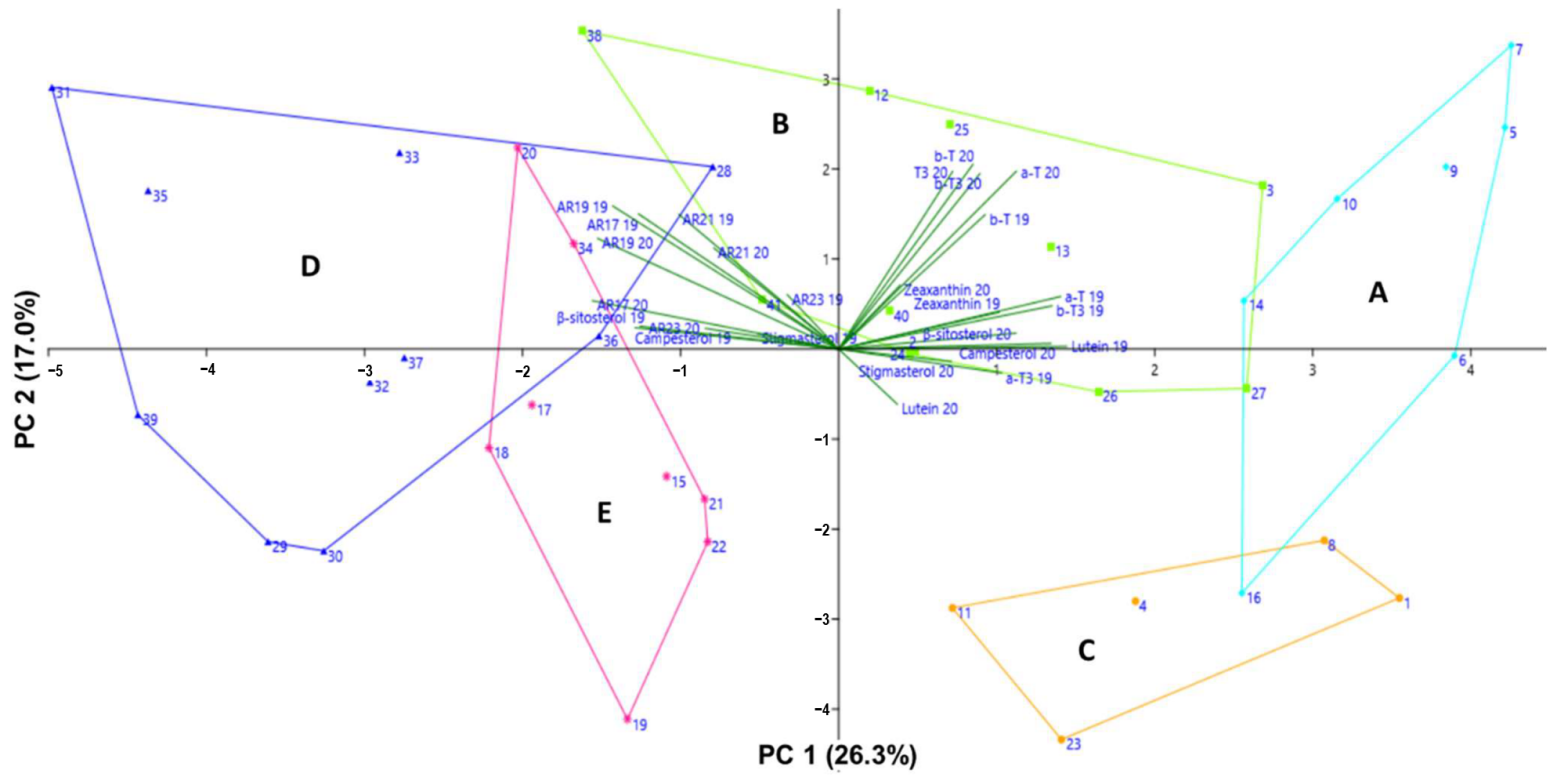
Principal component analysis (PCA) of the phytochemical profiles of 41 wheat cultivars. The PCA was performed on 13 phytochemical compounds measured in wheat cultivars harvested in 2019 and 2020. Each data point represents an individual cultivar, and cultivars are color-coded according to the clusters identified by hierarchical clustering analysis: Group A (cyan), Group B (green), Group C (orange), Group D (blue), and Group E (pink). The variables included in the PCA were alkylresorcinols (C17:0, heptadecylresorcinol; C19:0, nonadecylresorcinol; C21:0, heneicosylresorcinol; C23:0, tricosylresorcinol), phytosterols (campesterol, stigmasterol, and β-sitosterol), vitamin E homologues (α-T, α-tocopherol; α-T3, α-tocotrienol; β-T, β-tocopherol; β-T3, β-tocotrienol), and carotenoids (lutein and zeaxanthin). Variable labels followed by “19” and “20” indicate measurements obtained from samples harvested in 2019 and 2020, respectively. Numbers (1–41) indicate the cultivar codes corresponding to the wheat cultivars listed in Table 1.

PCA was employed to complement the hierarchical clustering results and to further elucidate relationships among metabolites. PC1 and PC2 explained 26.3% and 17.0% of the total variance, respectively (Figure 3). The PCA loading plot indicated a strong positive correlation between ARs and phytosterols, consistent with the clustering pattern observed in the heatmap, suggesting co-accumulation of these compounds across cultivars. Although ARs and phytosterols are synthesized via distinct metabolic pathways—polyketide for ARs and mevalonate for phytosterols [38,39]—their concurrent enrichment may be influenced by shared environmental influences or their distribution in similar grain fractions. Previous studies have also reported correlations among ARs, sterols, tocols, and other phytochemicals, likely reflecting differences in the relative contribution of the bran fraction [40].

Moderate positive correlations between carotenoids (lutein and zeaxanthin) and vitamin E homologs (α-tocopherol, β-tocopherol, and β-tocotrienol) were also observed. These compound classes share a common precursor, geranylgeranyl pyrophosphate (GGPP), which is synthesized in plastids. Carotenoids are produced through enzymatic reactions converting GGPP into downstream pigments such as lutein and zeaxanthin via the carotenoid biosynthetic pathway [41]. In contrast, vitamin E biosynthesis involves the conversion of GGPP to phytyl pyrophosphate, followed by further enzymatic transformations leading to the formation of tocopherols and tocotrienols [38]. This shared plastidial origin may partly explain the coordinated accumulation patterns observed among cultivars [42]. Overall, clustering analysis provides a useful tool for identifying wheat cultivars exhibiting superior phytochemical profiles with implications for improved health benefits.

### 3.5. Method Validation for Phytochemicals

All analytical methods for phytochemical quantification were validated in terms of precision and accuracy. Precision was evaluated through repeatability and reproducibility, and accuracy was assessed via recovery tests. As shown in Tables S9 and S10, repeatability CVs were below 8% for most compounds. Higher variability was observed for tocotrienols and β-tocopherol (CVs: 16.9–17.9%), likely due to their low concentrations in whole wheat. Reproducibility CVs remained below 8% across all analytes. Recovery rates exceeded 90% for all tested compounds—heptadecylresorcinol, β-sitosterol, α-tocopherol, and lutein—with CVs below 5%, indicating sufficient accuracy. In addition, the calibration curves for vitamin E homologues and carotenoids showed excellent linearity (R^2^ ≥ 0.997), and the limits of detection (LOD) and quantification (LOQ) are summarized in Table S2. These results confirm that the applied methods are suitable for the quantitative analysis of the target phytochemicals.

## 4. Conclusions

In conclusion, this study characterized the phytochemical profiles of 41 Korean wheat cultivars over two consecutive years, focusing on ARs, phytosterols, vitamin E, and carotenoids. AR levels remained relatively stable across years, suggesting that cultivar-related factors may contribute to their relative stability, whereas phytosterols and carotenoids showed greater interannual variation, indicating that their levels may be influenced by environmental conditions. Multivariate analyses, including principal component analysis (PCA) and hierarchical clustering, enabled the classification of cultivars based on phytochemical composition patterns. Notably, several cultivars were characterized by high contents of particular phytochemicals, whereas ‘Saekeumkang’ showed a consistently balanced profile across all compound groups. These findings highlight the potential of phytochemical profiling in guiding Korean wheat breeding programs. However, multi-year and multi-location studies are needed to validate genotype performance under diverse conditions.

## Figures and Tables

**Table 1:** List of 41 wheat cultivars.

No.	Cultivars	No.	Cultivars	No.	Cultivars	No.	Cultivars
1	Ol	11	Gobun	21	Jonong	31	Dajoong
2	Geuru	12	Keumkang	22	Jokyung	32	Goso
3	Dahong	13	Seodun	23	Yeonbaek	33	Joah
4	Chungkye	14	Saeol	24	Shinmichal 1	34	Hojoong
5	Eunpa	15	Jinpoom	25	Dabun	35	Baekchal
6	Tapdong	16	Milsung	26	Baekjoong	36	Jojoong
7	Namhae	17	Joeun	27	Jeokjoong	37	Baekkang
8	Uri	18	Anbaek	28	Sukang	38	Saekeumkang
9	Olgeuru	19	Jopoom	29	Hanbaek	39	Taejoong
10	Alchan	20	Shinmichal	30	Suan	40	Johan
						41	Hwanggeumal

**Table 2:** Descriptive statistics of phytochemical concentrations measured in 41 whole wheat cultivars harvested in 2019 and 2020.

		Min ^1)^	Max ^2)^	Mean ^3)^	SD ^4)^	Skewness ^5)^	Kurtosis ^6)^	CV (%) ^7)^
Phytochemicals (2019)								
Alkylresorcinols (mg/100 g)	Heptadecylresorcinol (C17:0)	1.21	3.15	2.19	0.49	0.11	–0.58	22.26
	Nonadecylresorcinol (C19:0)	10.57	25.74	17.55	3.78	0.2	–0.61	21.56
	Heneicosylresorcinol (C21:0)	17.97	34.38	25.81	4.09	–0.01	–0.35	15.86
	Tricosylresorcinol (C23:0)	5.3	11.86	9.02	1.64	–0.18	–0.69	18.21
Phytosterols (mg/100 g)	Campesterol	7.57	19	12.69	2.99	0.02	–1.02	23.59
	Stigmasterol	0.9	3.44	1.61	0.53	1.3	2.62	33.04
	β-Sitosterol	36.32	71.56	54.74	9.04	–0.11	–0.84	16.52
Vitamin E (mg/100 g)	α-Tocopherol	0.28	0.8	0.51	0.12	0.05	–0.29	22.77
	α-Tocotrienol	0.04	0.45	0.27	0.1	–0.45	–0.32	38.75
	β-Tocopherol	0.18	0.44	0.28	0.07	0.55	–0.25	23.58
	β-Tocotrienol	0.62	1.83	1.18	0.28	0.13	0.14	23.85
Carotenoids (μg/100 g)	Lutein	40.21	132.02	93.24	23.17	–0.64	–0.19	24.85
	Zeaxanthin	10.18	84.89	45.16	19.85	0.18	–1.17	43.96
Phytochemicals (2020)								
Alkylresorcinols (mg/100 g)	Heptadecylresorcinol (C17:0)	1.21	3.02	2.28	0.5	–0.53	–0.51	21.73
	Nonadecylresorcinol (C19:0)	11.46	24.22	17.48	2.87	0	–0.35	16.42
	Heneicosylresorcinol (C21:0)	17.86	35.14	24.91	3.35	0.82	1.73	13.45
	Tricosylresorcinol (C23:0)	8.02	16.31	12.05	2.26	–0.03	–0.60	18.73
Phytosterols (mg/100 g)	Campesterol	2.61	7.33	5	0.92	–0.10	0.36	18.45
	Stigmasterol	0.64	1.81	1.01	0.22	1.08	3.12	22.02
	β-Sitosterol	17.82	41.04	30.12	4.94	–0.73	0.8	16.42
Vitamin E (mg/100 g)	α-Tocopherol	0.42	1.4	0.77	0.24	0.58	–0.36	31
	α-Tocotrienol	0.06	0.43	0.25	0.09	0.04	–0.46	35.71
	β-Tocopherol	0.16	0.61	0.38	0.13	–0.17	–1.01	35.28
	β-Tocotrienol	0.67	2.9	1.73	0.7	–0.04	–1.34	40.51
Carotenoids (μg/100 g)	Lutein	41	98.85	67.3	13.39	0.06	–0.34	19.89
	Zeaxanthin	6.01	15.54	9.3	1.95	0.75	1.02	21

^1)^ Minimum content for each phytochemical. ^2)^ Maximum content for each phytochemical. ^3)^ Average content for each phytochemical. ^4)^ Standard deviation, which indicates dispersion of the phytochemical content from the mean. ^5)^ Asymmetry of the distribution of the phytochemical content. ^6)^ Measure of the tailedness of the probability distribution of the phytochemical content. ^7)^ Coefficient of variation, which represents the ratio of the standard deviation to the mean and is expressed as a percentage, indicating the relative variability of the phytochemical content.

**Table 3:** Comparison of average phytochemical concentrations among clusters (A–E) and between crop years (2019 vs. 2020) in 41 wheat cultivars.

		Groups (Number of Cultivars)				
		A (7)	B (11)	C (5)	D (10)	E (8)
Phytochemicals (2019)						
Alkylresorcinols (mg/100 g)	Heptadecylresorcinol (C17:0)	1.80 ± 0.39 ^bc^	2.31 ± 0.46 ^ab^	1.67 ± 0.20 ^c^	2.61 ± 0.36 ^a^	2.16 ± 0.34 ^abc^
	Nonadecylresorcinol (C19:0)	14.59 ± 2.45 ^bc^	18.12 ± 3.62 ^ab^	12.95 ± 1.67 ^c^	20.80 ± 2.92 ^a^	18.15 ± 2.36 ^ab^
	Heneicosylresorcinol (C21:0)	24.34 ± 3.14 ^a^	25.24 ± 3.98 ^a^	22.25 ± 4.20 ^a^	27.63 ± 3.69 ^a^	27.84 ± 4.00 ^a^
	Tricosylresorcinol (C23:0)	9.20 ± 1.26 ^a^	8.45 ± 1.79 ^a^	8.52 ± 2.44 ^a^	8.90 ± 1.50 ^a^	10.13 ± 1.02 ^a^
Phytosterols (mg/100 g)	Campesterol	11.35 ± 4.13 ^ab^	11.98 ± 2.40 ^ab^	9.23 ± 1.27 ^b^	14.74 ± 1.67 ^a^	14.43 ± 1.79 ^a^
	Stigmasterol	1.68 ± 0.64 ^ab^	1.37 ± 0.31 ^ab^	1.20 ± 0.16 ^b^	1.68 ± 0.36 ^ab^	2.02 ± 0.73 ^a^
	β-Sitosterol	53.18 ± 9.26 ^ab^	49.53 ± 8.07 ^b^	45.21 ± 5.72 ^b^	61.30 ± 5.09 ^a^	61.03 ± 5.65 ^a^
Vitamin E (mg/100 g)	α-Tocopherol	0.62 ± 0.08 ^a^	0.57 ± 0.11 ^a^	0.51 ± 0.03 ^a^	0.38 ± 0.07 ^b^	0.51 ± 0.09 ^a^
	α-Tocotrienol	0.29 ± 0.04 ^a^	0.34 ± 0.07 ^a^	0.29 ± 0.04 ^a^	0.14 ± 0.08 ^b^	0.30 ± 0.11 ^a^
	β-Tocopherol	0.37 ± 0.04 ^a^	0.29 ± 0.04 ^ab^	0.25 ± 0.04 ^b^	0.23 ± 0.04 ^b^	0.28 ± 0.09 ^b^
	β-Tocotrienol	1.39 ± 0.26 ^a^	1.22 ± 0.21 ^ab^	1.29 ± 0.33 ^a^	0.90 ± 0.21 ^b^	1.24 ± 0.21 ^ab^
Carotenoids (μg/100 g)	Lutein	99.70 ± 17.67 ^ab^	103.61 ± 12.82 ^ab^	120.13 ± 5.17 ^a^	63.19 ± 16.86 ^c^	94.09 ± 12.92 ^b^
	Zeaxanthin	62.02 ± 18.42 ^a^	47.74 ± 20.59 ^ab^	49.05 ± 26.45 ^ab^	27.61 ± 7.61 ^b^	46.37 ± 11.58 ^ab^
Phytochemicals (2020)						
Alkylresorcinols (mg/100 g)	Heptadecylresorcinol (C17:0)	1.71 ± 0.28 ^b^	2.44 ± 0.31 ^a^	1.72 ± 0.53 ^b^	2.58 ± 0.40 ^a^	2.54 ± 0.19 ^a,^*
	Nonadecylresorcinol (C19:0)	14.52 ± 1.45 ^c^	18.33 ± 1.93 ^ab^	13.86 ± 1.56 ^c^	20.36 ± 1.92 ^a^	17.57 ± 1.64 ^b^
	Heneicosylresorcinol (C21:0)	23.08 ± 1.91 ^a^	25.18 ± 3.20 ^a^	23.72 ± 2.49 ^a^	26.33 ± 3.67 ^a^	25.14 ± 4.26 ^a^
	Tricosylresorcinol (C23:0)	11.05 ± 2.35 ^a^	11.88 ± 2.34 ^a,^*	11.36 ± 2.75 ^a^	12.08 ± 2.02 ^a,^*	13.57 ± 1.72 ^a^
Phytosterols (mg/100 g)	Campesterol	6.11 ± 0.64 ^a,^*	4.85 ± 0.53 ^b,^*	5.40 ± 0.68 ^ab,^*	4.54 ± 1.12 ^b,^*	4.54 ± 0.56 ^b,^*
	Stigmasterol	1.22 ± 0.27 ^a^	0.94 ± 0.15 ^a,^*	0.97 ± 0.20 ^a^	0.94 ± 0.24 ^a,^*	1.02 ± 0.18 ^a,^*
	β-Sitosterol	35.29 ± 3.22 ^a,^*	29.41 ± 4.21 ^ab,^*	30.67 ± 1.89 ^ab,^*	28.17 ± 6.19 ^b,^*	28.65 ± 4.31 ^b,*^
Vitamin E (mg/100 g)	α-Tocopherol	1.10 ± 0.20 ^a,^*	0.90 ± 0.14 ^ab,^*	0.57 ± 0.11 ^c^	0.69 ± 0.14 ^bc,^*	0.55 ± 0.10 ^c^
	α-Tocotrienol	0.32 ± 0.09 ^a^	0.30 ± 0.04 ^a^	0.16 ± 0.07 ^b,^*	0.25 ± 0.08 ^ab,^*	0.17 ± 0.05 ^b,^*
	β-Tocopherol	0.50 ± 0.08 ^a,^*	0.45 ± 0.08 ^a,^*	0.24 ± 0.10 ^b^	0.34 ± 0.14 ^ab,^*	0.28 ± 0.09 ^b^
	β-Tocotrienol	2.27 ± 0.66 ^a,^*	2.23 ± 0.30 ^a,^*	1.17 ± 0.53 ^b^	1.65 ± 0.62 ^ab,^*	1.02 ± 0.38 ^b^
Carotenoids (μg/100 g)	Lutein	61.73 ± 11.39 ^b,^*	66.98 ± 12.06 ^b,^*	85.03 ± 10.55 ^a,^*	63.51 ± 13.09 ^b^	66.26 ± 11.98 ^b,^*
	Zeaxanthin	10.08 ± 1.46 ^a,^*	9.73 ± 2.41 ^a,^*	10.20 ± 1.75 ^a,^*	9.06 ± 1.82 ^a,^*	7.76 ± 1.18 ^a,^*

Cluster-wise comparison (within the same year): Different letters (a–c) in the same row indicate significant differences among clusters, as determined using Tukey’s honestly significant difference (HSD) test (*p* < 0.05). Year-wise comparison (within the same cluster): The * symbol next to 2020 values indicates a significant difference between 2019 and 2020 for the same compound within the same cluster, as determined using a two-tailed *t*-test (*p* < 0.05).

## Data Availability

The original contributions presented in this study are included in the article/Supplementary Materials. Further inquiries can be directed to the corresponding authors.

## Supplementary Materials

The following supporting information can be downloaded at: https://www.mdpi.com/article/10.3390/foods15061075/s1, Figure S1: Representative HPLC chromatograms used for the identification of carotenoids in whole wheat samples; Figure S2: Representative HPLC chromatograms used for the identification of vitamin E compounds in wheat samples; Figure S3: Representative GC chromatograms used for the identification of phytosterols in wheat samples; Figure S4: Representative GC chromatograms used for the identification of alkylresorcinols in wheat samples; Table S1. List of 41 wheat cultivars; Table S2: Calibration curves, coefficients of determination (R^2^), limits of detection (LOD), and limits of quantification (LOQ) for vitamin E homologues and carotenoids; Table S3: Alkylresorcinols (ARs) content identified in 41 wheat cultivars harvested in 2019 and 2020; Table S4: Phytosterols content identified in 41 wheat cultivars harvested in 2019 and 2020; Table S5: Vitamin E content identified in 41 wheat cultivars harvested in 2019 and 2020; Table S6: Carotenoids content identified in 41 wheat cultivars harvested in 2019 and 2020; Table S7: Climate conditions during the growth period in 2019 and 2020; Table S8: Two-way ANOVA results for the effects of cultivar, harvest year, and their interaction on phytochemical contents of whole wheat at the composite sample level; Table S9: Precision for the determination of phytochemicals in whole wheat; Table S10: Accuracy for the determination of phytochemicals in whole wheat; Table S11: Major classes of lipid-soluble phytochemicals analyzed in this study, including representative compounds and their reported biological and nutritional functions in wheat grains.
