# Supplementary material for: Analysis of Alkylresorcinol, Phytosterol, Carotenoid, and Vitamin E Contents in Korean Wheat Cultivars
Source: Foods. 2026 Mar 19;15(6):1075. doi: 10.3390/foods15061075 (PMC13024800; doi:10.3390/foods15061075)
Supplement: Supplementary file 1 [file foods-15-01075-s001.zip › foods-4164195-supplementary.pdf]

Supporting data

Analysis of Alkylresorcinol, Phytosterol, Carotenoid, and Vitamin E Contents in Korean Wheat Cultivars

Huijin Heo [1], Seonghwa Hong [1], Jinhee Park [2], Kyeong-Hoon Kim [2], Heon-Sang Jeong [1], Hana Lee [1,*] and Junsoo Lee [1,*]

[1] Department of Food Science and Biotechnology, Chungbuk National University, Cheongju 28644, Chungbuk, Republic of Korea; pltreasure11@gmail.com (H.H.); tjd3465@naver.com (S.H.); hsjeong@chungbuk.ac.kr (H.-S.J.)

[2] Wheat Research Team, National Institute of Crop Science, Wanju 55365, Jeonbuk, Republic of Korea; pjh237@korea.kr (J.P.); k2h0331@korea.kr (K.-H.K.)

* Correspondence: dlgksk0514@naver.com (H.L.); junsoo@chungbuk.ac.kr (J.L.)

Table S1. List of 41 wheat cultivars.

No.	Cultivars	Cross-combination
1	Ol	Norin 72/Norin 12
2	Geuru	F1(Strampelli/69D3607)/Chokwang
3	Dahong	Norin 72/Weongwang
4	Chungkye	Norin 4/Sharbatisonora
5	Eunpa	Chgoku 81/Tob-CNO/Yukseung3/Sw185
6	Tapdong	Chugoku 81//SW158/Toropi
7	Namhae	Olmil/Calidad
8	Uri	Geuru/Ol
9	Olgeuru	Geuru's'/Chokwang//Nishigai143
10	Alchan	Suwon210/Tapdong
11	Gobun	(Eunpa/Shannung6521)
12	Keumkang	F1Geuru‘S’/Kanto75//Eunpa
13	Seodun	Geurumil/Genaro 81
14	Saeol	Shirogane//F1(norin 43/Sonalika)
15	Jinpoom	Geurumil/Genaro81
16	Milsung	Sirogane//Norin 43/Sonalika
17	Joeun	Eunpamil/Suwon242
18	Anbaek	Saemil/Geurumil
19	Jopoom	SW88416-B-0/SW89277(F1)
20	Shinmichal	Olgeuru/kwandong 107/Baihuo
21	Jonong	SW234/SW80199-B-Y14-0
22	Jokyung	Seri82/Keumkang
23	Yeonbaek	Keumkang/Tapdong
24	Shinmichal 1	Alchan//Kanto107/Baihuo
25	Dabun	Suwon234//SW26039/Suwon220/3/Keumkang
26	Baekjoong	Keumkang/Olgeuru
27	Jeokjoong	Keumkang/Tapdong
28	Sukang	Suwon266/Asakaje
29	Hanbaek	Shann7859/Keumkang//Guamuehill
30	Suan	Keumkang/Eunpa//Keumkang
31	Dajoong	SW90149-B-1-SE3-3-2/Keumkang
32	Goso	IRENA/Olgeuru
33	Joah	SW86054-MB-27-3-2-1-1-1/Sumai#3
34	Hojoong	Alchan*2/3/Chunm18//JUP/BJY/4/Keumkang
35	Baekchal	Keumkang/Shinmichal
36	Jojoong	Suwon272/Olgeuru//Keumkang/Suwon252
37	Baekkang	Topdong/Klasic
38	Saekeumkang	Keumkang/Olgeuru
39	Taejoong	XIAN83(104).11/Keumkang
40	Johan	96PYT115/Suwon262//Joeun
41	Hwanggeumal	Jokyung/Suwon293

Table S2. Calibration curves, coefficients of determination (R^2), limits of detection (LOD), and limits of quantification (LOQ) for vitamin E homologues and carotenoids.

Standards	Regression equation	R^2	Calibration range (µg/mL)	Limit of detection (µg/mL)	Limit of quantification (µg/mL)
α-Tocopherol	y = 0.0000003x + 0.1367	0.9994	0.218–6.973	0.0690	0.0700
α-Tocotrienol	y = 0.0000004x + 0.016	0.9977	0.114–3.650	0.0522	0.0800
β-Tocopherol	y = 0.0000003x - 0.0204	0.9999	0.042–1.359	0.0150	0.0176
β-Tocotrienol	y = 0.0000003x + 0.0929	0.9999	0.063–2.019	0.0275	0.0280
ɣ-Tocopherol	y = 0.0000003x + 0.0857	0.9997	0.248–8.244	0.1087	0.1570
ɣ-Tocotrienol	y = 0.0000003x + 0.0354	0.9993	0.078–2.496	0.0365	0.0382
δ-Tocopherol	y = 0.0000002x + 0.0526	0.9996	0.115–3.680	0.0536	0.0552
δ-Tocotrienol	y = 0.0000002x + 0.0437	0.9996	0.066–2.124	0.0441	0.0446
Lutein	y = 0.000002x + 0.0518	0.9996	0.305–4.880	0.0039	0.0081
Zeaxanthin	y = 0.000006x + 0.0531	0.9997	0.215–3.440	0.0023	0.0033

Table S3. Alkylresorcinols (ARs) content identified in 41 wheat cultivars harvested in 2019 and 2020 (mg/100 g whole wheat)

No.	2019 AR(C17:0)	2019 AR (C19:0)	2019 AR (C21:0)	2019 AR (C23:0)	2020 AR (C17:0)	2020 AR (C19:0)	2020 AR (C21:0)	2020 AR (C23:0)
1	1.50 ± 0.01 ^{pqr}	10.57 ± 0.79 ^{p}	18.21 ± 1.20 ^{pq}	7.08 ± 0.17 ^{hijk}	1.21 ± 0.00 ^{m}	11.46 ± 0.01 ^{t}	20.07 ± 0.88 ^{mn}	8.15 ± 0.39 st
2	2.70 ± 0.05 ^{abcdef}	16.53 ± 0.02 ^{fghijklmn}	21.92 ± 0.04 ^{klmnopq}	8.40 ± 0.61 ^{cdefghijk}	2.36 ± 0.09 ^{abcdefghij}	18.84 ± 0.05 ^{defgh}	23.23 ± 0.80 ^{ghijklm}	8.38 ± 0.12 ^{rst}
3	2.24 ± 0.18 ^{efghijklm}	16.83 ± 0.56 ^{efghijklm}	21.21 ± 0.72 ^{mnopq}	7.44 ± 0.13 ^{fghijk}	2.16 ± 0.06 ^{cdefghijkl}	19.88 ± 0.04 ^{cdef}	24.71 ± 1.03 ^{defghijk}	9.10 ± 0.66 ^{pqrst}
4	1.93 ± 0.13 ^{hijklmnopq}	12.93 ± 0.87 ^{mnop}	22.48 ± 1.36 ^{jklmnopq}	8.79 ± 0.50 ^{abcdefghij}	1.39 ± 0.01 ^{lm}	14.37 ± 0.26 ^{nopqrs}	26.74 ± 1.76 ^{cdefg}	10.44 ± 0.54 ^{mnopqr}
5	2.07 ± 0.15 ^{ghijklmnop}	17.45 ± 0.26 ^{efghijkl}	26.26 ± 0.25 ^{cdefghijklmn}	9.73 ± 0.89 ^{abcdefghi}	1.61 ± 0.04 ^{jklm}	16.48 ± 0.55 ^{ijklm}	24.64 ± 1.03 ^{defghijk}	10.03 ± 0.39 ^{opqrst}
6	1.70 ± 0.15 ^{lmnopqr}	12.97 ± 0.23 ^{lmnop}	25.07 ± 0.36 ^{efghijklmno}	9.83 ± 0.25 ^{abcdefghi}	1.25 ± 0.07 ^{m}	12.44 ± 0.49 st	23.54 ± 1.16 ^{fghijklm}	8.63 ± 0.72 ^{qrst}
7	2.18 ± 0.15 ^{efghijklmn}	17.28 ± 1.19 ^{efghijklm}	28.06 ± 2.09 ^{cdefghij}	9.35 ± 0.87 ^{abcdefghi}	1.75 ± 0.13 ^{fghijklm}	15.85 ± 1.32 ^{klmno}	25.80 ± 2.39 ^{cdefghij}	10.86 ± 0.59 ^{lmnop}
8	1.83 ± 0.13 ^{ijklmnopq}	14.60 ± 0.48 ^{ijklmnop}	27.52 ± 1.07 ^{cdefghijk}	11.62 ± 0.49 ^{ab}	1.46 ± 0.24 ^{klm}	13.19 ± 0.09 ^{rst}	24.76 ± 0.80 ^{defghijk}	10.89 ± 0.09 ^{lmnop}
9	2.08 ± 0.16 ^{fghijklmnop}	16.59 ± 0.60 ^{fghijklmn}	24.67 ± 1.03 ^{efghijklmno}	7.43 ± 0.30 ^{fghijk}	1.64 ± 0.11 ^{ijklm}	15.30 ± 0.68 ^{lmnopq}	22.88 ± 0.35 ^{hijklm}	8.08 ± 0.12 st
10	1.98 ± 0.12 ^{hijklmnopq}	13.83 ± 0.19 ^{klmnop}	25.31 ± 0.50 ^{defghijklmno}	11.16 ± 0.21 ^{abcd}	1.66 ± 0.14 ^{hijklm}	13.39 ± 0.08 ^{qrst}	22.70 ± 0.05 ^{ijklm}	14.18 ± 0.92 ^{abcdef}
11	1.56 ± 0.03 ^{opqr}	14.43 ± 0.13 ^{jklmnop}	25.08 ± 0.15 ^{efghijklmno}	9.83 ± 0.75 ^{abcdefghi}	2.46 ± 0.61 ^{abcdefg}	15.09 ± 0.15 ^{lmnopqr}	24.25 ± 0.19 ^{efghijkl}	15.70 ± 0.92 ^{abcd}
12	2.89 ± 0.17 ^{abcd}	24.20 ± 0.09 ^{ab}	31.83 ± 0.19 ^{abc}	11.34 ± 0.04 ^{abcd}	2.78 ± 0.19 ^{abcd}	18.43 ± 0.24 ^{defghi}	22.21 ± 0.32 ^{jklm}	10.76 ± 0.68 ^{lmnopq}
13	1.81 ± 0.03 ^{jklmnopqr}	16.96 ± 0.15 ^{efghijklm}	27.75 ± 0.16 ^{cdefghij}	10.79 ± 0.84 ^{abcde}	2.23 ± 0.11 ^{bcdefghijk}	16.57 ± 0.41 ^{ijklm}	25.84 ± 0.55 ^{cdefghij}	13.82 ± 0.32 ^{cdefghi}
14	1.21 ± 0.05 ^{r}	11.99 ± 0.04 ^{op}	22.85 ± 0.03 ^{ijklmnopq}	9.08 ± 0.35 ^{abcdefghi}	2.15 ± 0.35 ^{cdefghijkl}	13.58 ± 0.74 ^{pqrs}	22.22 ± 1.38 ^{jklm}	13.70 ± 0.37 ^{defghij}
15	1.77 ± 0.04 ^{klmnopqr}	15.70 ± 0.23 ^{ghijklmno}	25.75 ± 0.40 ^{defghijklmn}	9.82 ± 1.65 ^{abcdefghi}	2.42 ± 0.04 ^{abcdefghi}	18.25 ± 0.49 ^{defghij}	27.71 ± 0.74 ^{cde}	16.13 ± 0.39 ^{ab}
16	1.36 ± 0.01 ^{qr}	12.02 ± 0.37 ^{op}	18.20 ± 0.51 ^{pq}	7.84 ± 0.48 ^{efghijk}	1.95 ± 0.03 ^{efghijklm}	14.61 ± 0.24 ^{mnopqr}	19.77 ± 0.36 ^{mn}	11.86 ± 0.36 ^{hijklmno}
17	2.14 ± 0.17 ^{efghijklmno}	18.33 ± 0.80 ^{defghijk}	29.57 ± 1.19 ^{abcdef}	10.53 ± 0.94 ^{abcdef}	2.71 ± 0.57 ^{abcde}	16.29 ± 0.27 ^{jklmn}	23.35 ± 0.81 ^{ghijklm}	13.97 ± 0.30 ^{bcdefgh}
18	2.35 ± 0.32 ^{cdefghijk}	17.71 ± 1.69 ^{efghijk}	27.77 ± 2.52 ^{cdefghij}	9.25 ± 1.24 ^{abcdefghi}	2.76 ± 0.13 ^{abcd}	18.31 ± 0.13 ^{defghi}	25.08 ± 0.38 ^{cdefghijk}	14.61 ± 0.03 ^{abcde}
19	1.60 ± 0.04 ^{nopqr}	14.00 ± 0.53 ^{jklmnop}	21.57 ± 0.75 ^{lmnopq}	9.91 ± 1.51 ^{abcdefgh}	2.44 ± 0.12 ^{abcdefgh}	14.08 ± 0.42 ^{opqrs}	17.86 ± 1.58 ^{n}	10.44 ± 0.47 ^{mnopqr}
20	2.45 ± 0.02 ^{cdefghi}	21.05 ± 1.99 ^{bcdef}	34.38 ± 0.10 ^{a}	11.86 ± 0.21 ^{a}	2.66 ± 0.50 ^{abcde}	18.94 ± 0.16 ^{defgh}	32.69 ± 0.43 ^{a}	14.12 ± 0.63 ^{abcdefg}
21	2.15 ± 0.01 ^{efghijklmno}	18.45 ± 0.76 ^{defghij}	28.69 ± 1.51 ^{bcdefgh}	10.53 ± 1.21 ^{abcdef}	2.52 ± 0.03 ^{abcdef}	17.75 ± 0.14 ^{ghijk}	24.74 ± 0.44 ^{defghijk}	13.19 ± 0.48 ^{efghijk}
22	2.64 ± 0.10 ^{abcdefg}	20.16 ± 0.31 ^{bcdefg}	24.23 ± 0.45 ^{efghijklmno}	8.48 ± 0.13 ^{bcdefghij}	2.62 ± 0.07 ^{abcde}	18.91 ± 0.38 ^{defgh}	23.05 ± 1.12 ^{ghijklm}	12.06 ± 0.08 ^{fghijklmno}
23	1.53 ± 0.02 ^{opqr}	12.23 ± 0.62 ^{nop}	17.97 ± 0.96 ^{q}	5.30 ± 0.22 ^{k}	2.08 ± 0.02 ^{defghijkl}	15.19 ± 0.76 ^{lmnopqr}	22.76 ± 1.12 ^{ijklm}	11.63 ± 0.06 ^{ijklmno}
24	1.61 ± 0.04 ^{mnopqr}	14.11 ± 0.47 ^{jklmnop}	26.60 ± 0.53 ^{cdefghijklm}	10.67 ± 0.15 ^{abcde}	2.14 ± 0.02 ^{cdefghijkl}	16.97 ± 0.55 ^{hijkl}	31.66 ± 0.93 ^{ab}	16.31 ± 0.12 ^{a}
25	2.69 ± 0.21 ^{abcdefg}	20.67 ± 0.89 ^{bcdef}	29.27 ± 1.37 ^{abcdefg}	8.35 ± 0.16 ^{cdefghijk}	2.77 ± 0.14 ^{abcd}	20.25 ± 0.54 ^{bcd}	28.74 ± 0.91 ^{bc}	13.39 ± 0.23 ^{efghijk}
26	1.91 ± 0.00 ^{hijklmnopq}	12.98 ± 0.31 ^{lmnop}	19.89 ± 0.50 ^{opq}	6.67 ± 0.10 ^{igk}	2.69 ± 0.12 ^{abcde}	18.03 ± 0.02 ^{fghij}	26.78 ± 0.00 ^{cdefg}	12.10 ± 0.53 ^{fghijklmno}
27	1.99 ± 0.19 ^{hijklmnop}	14.75 ± 1.25 ^{hijklmnop}	20.67 ± 2.55 ^{nopq}	5.79 ± 0.35 ^{jk}	2.19 ± 0.18 ^{cdefghijkl}	15.51 ± 0.62 ^{lmnop}	21.68 ± 0.80 ^{klmn}	10.46 ± 0.45 ^{mnopqr}
28	2.51 ± 0.35 ^{bcdefgh}	19.00 ± 2.45 ^{defghi}	23.65 ± 2.97 ^{ghijklmnop}	7.46 ± 1.66 ^{fghijk}	2.88 ± 0.00 ^{abc}	21.16 ± 0.05 ^{bc}	26.21 ± 0.13 ^{cdefghi}	12.97 ± 0.05 ^{efghijkl}
29	2.34 ± 0.32 ^{defghijk}	16.90 ± 2.76 ^{efghijklm}	25.12 ± 3.92 ^{defghijklmno}	7.62 ± 1.56 ^{efghijk}	3.02 ± 0.16 ^{a}	19.85 ± 0.61 ^{cdef}	26.82 ± 0.93 ^{cdefg}	11.92 ± 0.86 ^{ghijklmno}
30	2.25 ± 0.10 ^{efghijkl}	17.97 ± 2.08 ^{efghijk}	24.07 ± 2.41 ^{fghijklmno}	7.27 ± 0.70 ^{ghijk}	2.52 ± 0.07 ^{abcdefg}	20.14 ± 0.06 ^{bcde}	24.92 ± 0.05 ^{cdefghijk}	11.38 ± 1.36 ^{klmno}
31	3.10 ± 0.34 ^{ab}	25.74 ± 2.37 ^{a}	34.04 ± 2.04 ^{ab}	11.52 ± 1.38 ^{abc}	3.02 ± 0.04 ^{ab}	24.22 ± 0.02 ^{a}	35.14 ± 0.08 ^{a}	15.99 ± 0.10 ^{abc}
32	2.49 ± 0.06 ^{bcdefgh}	19.13 ± 1.69 ^{cdefgh}	27.20 ± 1.50 ^{cdefghijkl}	8.29 ± 0.33 ^{defghijk}	2.31 ± 0.03 ^{abcdefghij}	17.61 ± 0.31 ^{ghijk}	25.23 ± 0.44 ^{cdefghijk}	13.39 ± 0.55 ^{efghijk}
33	3.15 ± 0.21 ^{a}	24.29 ± 1.09 ^{ab}	31.88 ± 0.57 ^{abc}	9.81 ± 0.54 ^{abcdefghi}	2.75 ± 0.01 ^{abcd}	21.60 ± 0.73 ^{bc}	28.23 ± 1.16 ^{bcd}	12.29 ± 0.55 ^{fghijklmn}
34	2.16 ± 0.05 ^{efghijklmno}	19.84 ± 0.03 ^{bcdefg}	30.77 ± 0.28 ^{abcd}	10.65 ± 0.43 ^{abcde}	2.17 ± 0.10 ^{cdefghijkl}	17.99 ± 0.80 ^{ghijk}	26.67 ± 1.27 ^{cdefgh}	14.08 ± 0.15 ^{bcdefg}
35	2.96 ± 0.01 ^{abc}	23.64 ± 0.77 ^{abc}	28.16 ± 0.90 ^{cdefghi}	8.30 ± 0.52 ^{defghijk}	2.85 ± 0.06 ^{abcd}	21.66 ± 0.22 ^{bc}	25.24 ± 0.13 ^{cdefghijk}	12.39 ± 0.20 ^{fghijklmn}
36	2.08 ± 0.13 ^{efghijklmnop}	19.22 ± 0.09 ^{cdefgh}	29.85 ± 0.36 ^{abcde}	10.35 ± 0.02 ^{abcdefg}	1.73 ± 0.02 ^{ghijklm}	20.21 ± 0.48 ^{bcd}	24.32 ± 0.34 ^{efghijkl}	8.02 ± 0.28 ^{t}
37	2.73 ± 0.04 ^{abcde}	21.14 ± 0.19 ^{bcde}	23.08 ± 0.22 ^{hijklmnopq}	7.92 ± 0.18 efghijk	2.35 ± 0.04 ^{abcdefghij}	18.15 ± 0.25 ^{efghij}	20.72 ± 0.22 ^{lmn}	10.88 ± 0.51 ^{lmnop}
38	2.97 ± 0.09 ^{abc}	22.83 ± 1.41 ^{abcd}	28.02 ± 0.45 ^{cdefghij}	7.18 ± 0.26 ^{ghijk}	3.01 ± 0.05 ^{ab}	22.10 ± 1.00 ^{b}	27.29 ± 1.66 ^{cdef}	13.59 ± 0.92 ^{defghijk}
39	2.46 ± 0.03 ^{cdefgh}	20.97 ± 0.65 ^{bcdef}	29.22 ± 1.13 ^{abcdefg}	10.47 ± 0.36 ^{abcdef}	2.40 ± 0.08 ^{abcdefghij}	19.03 ± 0.35 ^{defg}	26.41 ± 0.75 ^{cdefghi}	11.54 ± 0.91 ^{jklmno}
40	2.16 ± 0.10 ^{efghijklmno}	19.16 ± 0.93 ^{cdefgh}	27.04 ± 1.65 ^{cdefghijkl}	8.37 ± 0.76 ^{cdefghijk}	2.15 ± 0.23 ^{cdefghijkl}	16.43 ± 0.38 ^{ijklm}	22.68 ± 0.68 ^{ijklm}	10.28 ± 0.12 ^{nopqrs}
41	2.40 ± 0.16 ^{cdefghij}	20.30 ± 0.36 ^{bcdef}	23.46 ± 0.51 ^{hijklmnopq}	7.89 ± 0.81 ^{efghijk}	2.39 ± 0.10 ^{abcdefghij}	18.65 ± 0.41 ^{defgh}	22.15 ± 0.57 ^{jklm}	12.50 ± 0.08 ^{efghijklm}

Values are the mean ± standard error (n = 2). Statistical analysis was performed using one-way analysis of variance (ANOVA) followed by Tukey's multiple comparison test to compare the 41 cultivars for each component, without considering the year factor. Different letters within a column indicate significant differences among cultivars ($p < 0.05$). AR (C17:0), 5-*n*-Heptadecylresorcinol; AR (C19:0), 5-*n*-Nonadecylresorcinol; AR (C21:0), 5-*n*-Heneicosylresorcinol; AR (C23:0), 5-*n*-Tricosylresorcinol.

Table S4. Phytosterols content identified in 41 wheat cultivars harvested in 2019 and 2020

(mg/100 g whole wheat)

	2019			2020		
No.	Campesterol	Stigmasterol	β-sitosterol	Campesterol	Stigmasterol	β-sitosterol
1	9.43 ± 0.29 ^{fghijkl}	1.25 ± 0.01 ^{bc}	47.48 ± 1.75 ^{hijklm}	4.52 ± 0.24 ^{bcdefg}	1.01 ± 0.09 ^{ab}	28.06 ± 1.35 ^{bcdefgh}
2	9.04 ± 0.37 ^{hijkl}	1.08 ± 0.00 ^{bc}	41.25 ± 0.38 ^{jklm}	3.95 ± 0.13 ^{cdefg}	0.85 ± 0.04 ^{b}	25.05 ± 1.21 ^{cdefgh}
3	9.54 ± 0.59 ^{efghijkl}	0.9 ± 0.04 ^{c}	49.26 ± 1.90 ^{ghijkl}	5.10 ± 0.42 ^{abcdef}	0.89 ± 0.03 ^{ab}	35.31 ± 1.16 ^{abc}
4	8.41 ± 0.25 ^{jkl}	1.02 ± 0.02 ^{bc}	40.52 ± 1.89 ^{klm}	4.98 ± 0.01 ^{bcdef}	0.64 ± 0.23 ^{b}	33.14 ± 0.51 ^{abcd}
5	8.72 ± 0.32 ^{ijkl}	1.72 ± 0.21 ^{abc}	50.01 ± 1.62 ^{fghijkl}	6.12 ± 0.39 ^{abcd}	1.08 ± 0.03 ^{ab}	36.38 ± 0.02 ^{ab}
6	7.57 ± 0.50 ^{l}	1.35 ± 0.12 ^{bc}	42.25 ± 1.86 ^{jklm}	6.38 ± 0.10 ^{ab}	1.09 ± 0.05 ^{ab}	36.15 ± 1.23 ^{abc}
7	9.53 ± 0.38 ^{efghijkl}	1.54 ± 0.23 ^{abc}	49.74 ± 1.06 ^{fghijkl}	6.21 ± 0.11 ^{abc}	1.02 ± 0.04 ^{ab}	34.34 ± 0.15 ^{abc}
8	8.35 ± 1.25 ^{kl}	1.45 ± 0.14 ^{bc}	39.27 ± 1.80 ^{lm}	6.00 ± 0.10 ^{abcd}	1.15 ± 0.00 ^{ab}	31.00 ± 0.09 ^{abcde}
9	10.19 ± 0.02 ^{defghijkl}	1.23 ± 0.09 ^{bc}	51.12 ± 2.66 ^{efghijkl}	5.40 ± 0.06 ^{abcdef}	1.25 ± 0.02 ^{ab}	35.55 ± 1.18 ^{abc}
10	9.32 ± 0.56 ^{ghijkl}	1.00 ± 0.29 ^{c}	49.89 ± 0.41 ^{fghijkl}	5.67 ± 0.24 ^{abcde}	1.07 ± 0.03 ^{ab}	32.67 ± 0.64 ^{abcd}
11	11.37 ± 0.71 ^{cdefghijkl}	1.19 ± 0.09 ^{bc}	45.27 ± 2.46 ^{ijklm}	6.15 ± 0.15 ^{abcd}	1.10 ± 0.07 ^{ab}	31.37 ± 1.00 ^{abcde}
12	9.69 ± 0.22 ^{efghijkl}	1.10 ± 0.01 ^{bc}	49.33 ± 0.38 ^{ghijkl}	4.60 ± 0.29 ^{bcdefg}	1.06 ± 0.04 ^{ab}	33.24 ± 1.20 ^{abcd}
13	13.86 ± 1.54 ^{abcdefghijk}	1.55 ± 0.77 ^{abc}	51.49 ± 7.07 ^{efghijkl}	5.52 ± 0.03 ^{abcdef}	0.88 ± 0.07 ^{ab}	31.24 ± 1.17 ^{abcde}
14	19.00 ± 1.10 ^{a}	2.92 ± 0.07 ^{ab}	71.56 ± 0.51 ^{a}	7.33 ± 0.20 ^{a}	1.19 ± 0.03 ^{ab}	41.04 ± 1.01 ^{a}
15	17.30 ± 5.14 ^{ab}	3.44 ± 2.23 ^{a}	55.85 ± 6.95 ^{bcdefghi}	5.30 ± 0.05 ^{abcdef}	1.11 ± 0.29 ^{ab}	28.82 ± 2.96 ^{bcdefg}
16	15.11 ± 0.14 ^{abcde}	2.00 ± 0.03 ^{abc}	57.68 ± 0.01 ^{bcdefghi}	5.63 ± 0.13 ^{abcde}	1.81 ± 0.66 ^{a}	30.90 ± 0.39 ^{abcdef}
17	12.64 ± 0.50 ^{bcdefghijkl}	1.77 ± 0.07 ^{abc}	63.47 ± 2.50 ^{abcde}	4.22 ± 0.17 ^{bcdefg}	0.71 ± 0.07 ^{b}	30.84 ± 1.38 ^{abcdef}
18	14.29 ± 0.68 ^{abcdefghi}	1.86 ± 0.20 ^{abc}	68.38 ± 0.45 ^{ab}	4.42 ± 0.15 ^{bcdefg}	0.96 ± 0.16 ^{ab}	26.43 ± 0.22 ^{bcdefgh}
19	15.90 ± 0.86 ^{abc}	2.13 ± 0.43 ^{abc}	61.03 ± 0.34 ^{abcdefg}	3.92 ± 0.21 ^{defg}	0.90 ± 0.04 ^{ab}	25.71 ± 1.78 ^{bcdefgh}
20	15.74 ± 0.40 ^{abcd}	2.35 ± 0.01 ^{abc}	65.40 ± 0.49 ^{abc}	4.20 ± 0.20 ^{bcdefg}	0.98 ± 0.39 ^{ab}	20.74 ± 1.50 ^{efgh}
21	12.51 ± 0.39 ^{bcdefghijkl}	1.29 ± 0.02 ^{bc}	61.36 ± 0.76 ^{abcdefg}	4.95 ± 0.10 ^{bcdef}	1.11 ± 0.02 ^{ab}	34.06 ± 0.47 ^{abc}
22	12.61 ± 0.11 ^{bcdefghijkl}	1.08 ± 0.08 ^{bc}	50.28 ± 0.05 ^{fghijkl}	5.28 ± 0.12 ^{abcdef}	1.33 ± 0.44 ^{ab}	32.96 ± 1.36 ^{abcd}
23	8.59 ± 3.28 ^{jkl}	1.11 ± 0.48 ^{bc}	53.49 ± 0.45 ^{cdefghij}	5.34 ± 0.04 ^{abcdef}	0.97 ± 0.06 ^{ab}	29.75 ± 0.54 ^{bcdefg}
24	11.44 ± 0.45 ^{cdefghijkl}	1.25 ± 0.23 ^{bc}	42.16 ± 1.33 ^{jklm}	5.50 ± 0.98 ^{abcdef}	0.84 ± 0.24 ^{b}	19.79 ± 1.20 ^{fgh}
25	12.61 ± 0.23 ^{bcdefghijkl}	1.79 ± 0.25 ^{abc}	53.47 ± 0.20 ^{cdefghij}	5.01 ± 0.84 ^{bcdef}	1.33 ± 0.32 ^{ab}	31.75 ± 3.54 ^{abcde}
26	12.49 ± 0.02 ^{bcdefghijkl}	1.39 ± 0.06 ^{bc}	46.74 ± 1.02 ^{hijklm}	5.03 ± 0.01 ^{bcdef}	0.93 ± 0.07 ^{ab}	29.00 ± 1.04 ^{bcdefgh}
27	9.35 ± 1.76 ^{ghijkl}	1.10 ± 0.11 ^{bc}	36.32 ± 4.18 ^{m}	4.62 ± 0.16 ^{bcdefg}	0.91 ± 0.01 ^{ab}	28.14 ± 0.04 ^{bcdefgh}
28	14.41 ± 0.71 ^{abcdefgh}	1.99 ± 0.16 ^{abc}	58.30 ± 3.26 ^{bcdefgh}	5.77 ± 0.41 ^{abcde}	1.29 ± 0.30 ^{ab}	31.77 ± 1.29 ^{abcde}
29	13.42 ± 0.06 ^{abcdefghijk}	1.63 ± 0.38 ^{abc}	66.84 ± 1.45 ^{ab}	5.68 ± 0.01 ^{abcde}	1.22 ± 0.42 ^{ab}	31.76 ± 0.12 ^{abcde}
30	11.53 ± 0.62 ^{cdefghijkl}	1.12 ± 0.17 ^{bc}	61.10 ± 0.42 ^{abcdefg}	3.71 ± 0.33 ^{efg}	0.95 ± 0.09 ^{ab}	31.40 ± 3.04 ^{abcde}
31	14.80 ± 0.15 ^{abcdefg}	1.00 ± 0.18 ^{c}	55.82 ± 1.11 ^{bcdefghi}	4.01 ± 2.14 ^{cdefg}	0.83 ± 0.50 ^{b}	22.49 ± 4.04 ^{defgh}
32	15.94 ± 0.15 ^{abc}	1.91 ± 0.39 ^{abc}	67.41 ± 4.87 ^{ab}	4.61 ± 0.09 ^{bcdefg}	0.71 ± 0.00 ^{b}	29.84 ± 0.45 ^{bcdefg}
33	14.87 ± 0.26 ^{abcdefg}	2.03 ± 0.28 ^{abc}	66.51 ± 5.22 ^{ab}	5.55 ± 0.08 ^{abcdef}	1.20 ± 0.04 ^{ab}	36.20 ± 0.28 ^{abc}
34	14.44 ± 0.14 ^{abcdefgh}	2.27 ± 0.42 ^{abc}	62.45 ± 0.27 ^{abcdef}	4.04 ± 0.08 ^{cdefg}	1.06 ± 0.01 ^{ab}	29.62 ± 0.95 ^{bcdefg}
35	13.99 ± 2.18 ^{abcdefghij}	1.72 ± 0.34 ^{abc}	53.25 ± 9.79 ^{cdefghijk}	3.36 ± 0.11 ^{fg}	0.70 ± 0.03 ^{b}	17.82 ± 1.14 ^{h}
36	16.75 ± 0.50 ^{abc}	1.77 ± 0.04 ^{abc}	62.54 ± 1.00 ^{abcdef}	5.68 ± 0.08 ^{abcde}	1.01 ± 0.02 ^{ab}	31.99 ± 0.65 ^{abcd}
37	17.32 ± 2.93 ^{ab}	1.98 ± 0.05 ^{abc}	64.58 ± 3.81 ^{abcd}	4.41 ± 0.17 ^{bcdefg}	0.80 ± 0.02 ^{b}	29.50 ± 0.95 ^{bcdefg}
38	16.05 ± 2.62 ^{abc}	1.83 ± 0.68 ^{abc}	65.73 ± 4.87 ^{abc}	4.24 ± 0.07 ^{bcdefg}	0.82 ± 0.04 ^{b}	28.95 ± 0.06 ^{bcdefgh}
39	14.34 ± 0.15 ^{abcdefgh}	1.71 ± 0.04 ^{abc}	56.67 ± 3.44 ^{bcdefghi}	2.61 ± 2.14 ^{g}	0.65 ± 0.57 ^{b}	18.93 ± 14.59 ^{gh}
40	15.00 ± 0.07 ^{abcdef}	1.67 ± 0.49 ^{abc}	57.26 ± 0.01 ^{bcdefghi}	5.43 ± 0.05 ^{abcdef}	0.85 ± 0.19 ^{b}	31.78 ± 0.11 ^{abcde}
41	12.72 ± 1.36 ^{bcdefghijkl}	1.47 ± 0.80 ^{bc}	51.81 ± 2.99 ^{defghijkl}	4.38 ± 0.13 ^{bcdefg}	0.97 ± 0.02 ^{ab}	29.24 ± 0.48 ^{bcdefg}

Values are the mean ± standard error ($n = 2$).

Statistical analysis was performed using one-way analysis of variance (ANOVA) followed by Tukey's multiple comparison test to compare the 41 cultivars for each component, without considering the year factor. Different letters within a column indicate significant differences among cultivars ($p < 0.05$).

Table S5. Vitamin E content identified in 41 wheat cultivars harvested in 2019 and 2020

(mg/100 g whole wheat)

	2019				2020			
No.	α-Tocopherol	α-Tocotrienol	β-Tocopherol	β-Tocotrienol	α-Tocopherol	α-Tocotrienol	β-Tocopherol	β-Tocotrienol
1	0.53 ± 0.01 ^{cdefghi}	0.26 ± 0.00 ^{hijklm}	0.25 ± 0.00 ^{ghijklmn}	1.40 ± 0.02 ^{bcdefg}	0.75 ± 0.06 ^{defghijkl}	0.26 ± 0.02 ^{defghi}	0.39 ± 0.04 ^{defghijk}	2.10 ± 0.20 ^{bcdefghi}
2	0.52 ± 0.05 ^{defghij}	0.27 ± 0.00 ^{hijklm}	0.26 ± 0.03 ^{fghijklmn}	1.42 ± 0.02 ^{bcdef}	0.75 ± 0.20 ^{defghijkl}	0.27 ± 0.07 ^{cdefgh}	0.41 ± 0.09 ^{cdefghi}	2.41 ± 0.79 ^{abcdef}
3	0.58 ± 0.00 ^{bcdefg}	0.30 ± 0.00 ^{fghij}	0.29 ± 0.01 ^{defghijkl}	1.53 ± 0.00 ^{abcd}	1.11 ± 0.05 ^{abc}	0.41 ± 0.04 ^{ab}	0.51 ± 0.02 ^{abcde}	2.75 ± 0.17 ^{abc}
4	0.53 ± 0.02 ^{defghij}	0.27 ± 0.00 ^{hijklm}	0.26 ± 0.01 ^{fghijklmn}	1.26 ± 0.01 ^{cdefghijk}	0.63 ± 0.01 ^{ijklmn}	0.17 ± 0.00 ^{hijklm}	0.23 ± 0.00 ^{klmn}	1.01 ± 0.01 ^{mnop}
5	0.65 ± 0.03 ^{bcd}	0.25 ± 0.02 ^{ijklmn}	0.39 ± 0.01 ^{abc}	1.83 ± 0.10 ^{a}	1.18 ± 0.04 ^{ab}	0.32 ± 0.00 ^{bcde}	0.51 ± 0.02 ^{abcdef}	2.63 ± 0.07 ^{abcd}
6	0.49 ± 0.04 ^{efghijklm}	0.24 ± 0.00 ^{klmno}	0.33 ± 0.02 ^{cdefgh}	1.20 ± 0.04 ^{efghijkl}	1.02 ± 0.11 ^{bcdef}	0.28 ± 0.01 ^{cdefg}	0.49 ± 0.03 ^{abcdefg}	1.83 ± 0.07 ^{fghijk}
7	0.69 ± 0.06 ^{ab}	0.24 ± 0.01 ^{klmno}	0.44 ± 0.00 ^{a}	1.66 ± 0.08 ^{ab}	1.40 ± 0.14 ^{a}	0.31 ± 0.02 ^{cde}	0.61 ± 0.04 ^{a}	2.62 ± 0.11 ^{abcde}
8	0.53 ± 0.01 ^{defghij}	0.30 ± 0.01 ^{fghij}	0.31 ± 0.02 ^{cdefghij}	1.78 ± 0.17 ^{a}	0.49 ± 0.10 ^{klmn}	0.17 ± 0.03 ^{hijklm}	0.16 ± 0.03 ^{n}	1.08 ± 0.21 ^{lmnop}
9	0.61 ± 0.05 ^{bcde}	0.33 ± 0.02 ^{cdefg}	0.37 ± 0.02 ^{abcd}	1.22 ± 0.06 ^{defghijk}	1.14 ± 0.00 ^{abc}	0.43 ± 0.01 ^{a}	0.53 ± 0.01 ^{abcd}	2.82 ± 0.12 ^{ab}
10	0.54 ± 0.00 ^{cdefghi}	0.31 ± 0.00 ^{efghi}	0.32 ± 0.00 ^{cdefgh}	1.18 ± 0.00 ^{efghijk}	1.12 ± 0.08 ^{abc}	0.42 ± 0.01 ^{ab}	0.50 ± 0.05 ^{abcdef}	2.90 ± 0.04 ^{a}
11	0.46 ± 0.10 ^{ghijklmn}	0.27 ± 0.01 ^{ghijkl}	0.25 ± 0.04 ^{ghijklmn}	1.01 ± 0.02 ^{jklm}	0.50 ± 0.03 ^{klmn}	0.13 ± 0.00 ^{jklm}	0.16 ± 0.01 ^{n}	0.82 ± 0.03 ^{op}
12	0.57 ± 0.02 ^{bcdefgh}	0.27 ± 0.00 ^{ghijkl}	0.33 ± 0.01 ^{cdefgh}	1.15 ± 0.01 ^{efghijkl}	1.03 ± 0.06 ^{bcd}	0.30 ± 0.01 ^{cde}	0.45 ± 0.03 ^{abcdefgh}	2.34 ± 0.08 ^{abcdefg}
13	0.61 ± 0.03 ^{bcdef}	0.32 ± 0.01 ^{efgh}	0.31 ± 0.02 ^{cdefghi}	1.56 ± 0.08 ^{abc}	1.02 ± 0.09 ^{bcde}	0.30 ± 0.05 ^{cde}	0.40 ± 0.03 ^{defghij}	2.31 ± 0.33 ^{abcdefgh}
14	0.67 ± 0.02 ^{abc}	0.33 ± 0.02 ^{defgh}	0.39 ± 0.01 ^{abc}	1.30 ± 0.06 ^{cdefghij}	1.11 ± 0.18 ^{abc}	0.30 ± 0.04 ^{cde}	0.51 ± 0.09 ^{abcdef}	2.02 ± 0.32 ^{cdefghi}
15	0.53 ± 0.02 ^{defghij}	0.33 ± 0.01 ^{defgh}	0.25 ± 0.00 ^{ghijklmn}	1.39 ± 0.08 ^{bcdefg}	0.57 ± 0.03 ^{jklmn}	0.17 ± 0.00 ^{hijklm}	0.28 ± 0.01 ^{ijklmn}	1.02 ± 0.01 ^{mnop}
16	0.69 ± 0.02 ^{ab}	0.31 ± 0.01 ^{efgh}	0.34 ± 0.00 ^{cdefg}	1.33 ± 0.07 ^{cdefghi}	0.72 ± 0.06 ^{ghijklmn}	0.18 ± 0.01 ^{ghijkl}	0.34 ± 0.03 ^{ghijklm}	1.07 ± 0.07 ^{mnop}
17	0.51 ± 0.00 ^{defghijkl}	0.22 ± 0.00 ^{lmno}	0.36 ± 0.01 ^{abcde}	1.38 ± 0.09 ^{bcdefg}	0.61 ± 0.08 ^{ijklmn}	0.13 ± 0.01 ^{jklm}	0.31 ± 0.06 ^{hijklmn}	0.85 ± 0.11 ^{nop}
18	0.43 ± 0.03 ^{hijklmno}	0.25 ± 0.01 ^{ijklmn}	0.24 ± 0.00 ^{hijklmn}	1.37 ± 0.13 ^{bcdefgh}	0.56 ± 0.07 ^{jklmn}	0.17 ± 0.02 ^{hijklm}	0.24 ± 0.03 ^{jklmn}	1.13 ± 0.12 ^{klmnop}
19	0.35 ± 0.00 ^{nop}	0.23 ± 0.00 ^{klmno}	0.21 ± 0.03 ^{jklmn}	0.99 ± 0.05 ^{klm}	0.43 ± 0.12 ^{mn}	0.10 ± 0.02 ^{lm}	0.17 ± 0.04 ^{n}	0.67 ± 0.11 ^{p}
20	0.57 ± 0.04 ^{bcdefgh}	0.41 ± 0.03 ^{ab}	0.44 ± 0.03 ^{ab}	1.44 ± 0.08 ^{bcde}	0.65 ± 0.03 ^{ijklmn}	0.25 ± 0.00 ^{defghi}	0.40 ± 0.00 ^{defghij}	1.15 ± 0.01 ^{jklmnop}
21	0.63 ± 0.01 ^{bcd}	0.41 ± 0.01 ^{ab}	0.24 ± 0.00 ^{hijklmn}	1.09 ± 0.02 ^{ghijklm}	0.42 ± 0.02 ^{n}	0.15 ± 0.02 ^{ijklm}	0.21 ± 0.01 ^{lmn}	0.76 ± 0.01 ^{p}
22	0.53 ± 0.00 ^{defghij}	0.40 ± 0.00 ^{ab}	0.18 ± 0.00 ^{mn}	0.90 ± 0.01 ^{lmno}	0.47 ± 0.06 ^{lmn}	0.16 ± 0.03 ^{ijklm}	0.21 ± 0.03 ^{lmn}	0.72 ± 0.06 ^{p}
23	0.52 ± 0.01 ^{defghijk}	0.36 ± 0.00 ^{bcdef}	0.20 ± 0.02 ^{klmn}	0.97 ± 0.00 ^{klmn}	0.49 ± 0.00 ^{klmn}	0.06 ± 0.09 ^{m}	0.28 ± 0.00 ^{ijklmn}	0.84 ± 0.02 ^{op}
24	0.62 ± 0.01 ^{bcde}	0.45 ± 0.01 ^{a}	0.23 ± 0.00 ^{ijklmn}	1.09 ± 0.02 ^{ghijkl}	0.78 ± 0.03 ^{defghijk}	0.33 ± 0.03 ^{abcde}	0.37 ± 0.01 ^{efghijkl}	1.55 ± 0.16 ^{ijklmno}
25	0.80 ± 0.02 ^{a}	0.39 ± 0.00 ^{bcd}	0.34 ± 0.01 ^{cdefg}	1.20 ± 0.03 ^{efghijkl}	0.96 ± 0.01 ^{bcdefgh}	0.26 ± 0.01 ^{defghi}	0.57 ± 0.00 ^{ab}	2.00 ± 0.04 ^{defghi}
26	0.61 ± 0.02 ^{bcdef}	0.41 ± 0.00 ^{ab}	0.25 ± 0.00 ^{ghijklmn}	1.31 ± 0.04 ^{defghij}	0.70 ± 0.04 ^{ghijklmn}	0.30 ± 0.00 ^{cde}	0.42 ± 0.02 ^{bcdefghi}	2.24 ± 0.01 ^{abcdefghi}
27	0.64 ± 0.03 ^{bcd}	0.39 ± 0.01 ^{bc}	0.25 ± 0.02 ^{fghijklmn}	1.15 ± 0.11 ^{efghijkl}	0.76 ± 0.01 ^{defghijkl}	0.29 ± 0.00 ^{cde}	0.57 ± 0.14 ^{abc}	2.39 ± 0.12 ^{abcdef}
28	0.42 ± 0.00 ^{ijklmnop}	0.11 ± 0.00 ^{qr}	0.22 ± 0.00 ^{jklmn}	1.11 ± 0.01 ^{fghijkl}	0.96 ± 0.06 ^{bcdefgh}	0.37 ± 0.01 ^{abc}	0.57 ± 0.03 ^{ab}	2.79 ± 0.35 ^{ab}
29	0.37 ± 0.01 ^{mnop}	0.07 ± 0.00 ^{rs}	0.18 ± 0.01 ^{n}	0.66 ± 0.02 ^{no}	0.56 ± 0.01 ^{jklmn}	0.16 ± 0.00 ^{ijklm}	0.16 ± 0.00 ^{n}	0.78 ± 0.01 ^{p}
30	0.38 ± 0.01 ^{klmnop}	0.05 ± 0.00 ^{rs}	0.20 ± 0.01 ^{lmn}	0.88 ± 0.01 ^{lmno}	0.57 ± 0.00 ^{jklmn}	0.12 ± 0.00 ^{klm}	0.16 ± 0.01 ^{n}	0.87 ± 0.04 ^{nop}
31	0.43 ± 0.01 ^{ijklmno}	0.04 ± 0.00 ^{s}	0.22 ± 0.01 ^{jklmn}	1.04 ± 0.06 ^{ijklm}	0.81 ± 0.02 ^{defghij}	0.23 ± 0.00 ^{efghij}	0.39 ± 0.00 ^{defghijk}	2.03 ± 0.01 ^{cdefghi}
32	0.39 ± 0.06 ^{jklmnop}	0.11 ± 0.02 ^{qr}	0.22 ± 0.02 ^{ijklmn}	1.11 ± 0.09 ^{fghijkl}	0.61 ± 0.02 ^{ijklmn}	0.23 ± 0.00 ^{efghij}	0.34 ± 0.01 ^{ghijklm}	1.59 ± 0.01 ^{hijklmn}
33	0.54 ± 0.02 ^{cdefghi}	0.14 ± 0.01 ^{pq}	0.27 ± 0.00 ^{efghijklm}	1.16 ± 0.05 ^{efghijkl}	0.71 ± 0.02 ^{ghijklmn}	0.29 ± 0.00 ^{cdef}	0.38 ± 0.01 ^{defghijk}	1.82 ± 0.01 ^{fghijkl}
34	0.53 ± 0.06 ^{defghij}	0.13 ± 0.01 ^{pq}	0.33 ± 0.02 ^{cdefg}	1.32 ± 0.10 ^{cdefghij}	0.68 ± 0.04 ^{hijklmn}	0.25 ± 0.01 ^{defghi}	0.44 ± 0.02 ^{bcdefghi}	1.82 ± 0.09 ^{fghijk}
35	0.31 ± 0.02 ^{op}	0.18 ± 0.02 ^{op}	0.27 ± 0.04 ^{efghijklm}	0.62 ± 0.11 ^{o}	0.73 ± 0.03 ^{efghijklm}	0.30 ± 0.00 ^{cde}	0.48 ± 0.02 ^{abcdefg}	1.60 ± 0.04 ^{ghijklm}
36	0.33 ± 0.00 ^{op}	0.19 ± 0.01 ^{nop}	0.27 ± 0.01 ^{efghijklmn}	1.00 ± 0.12 ^{jklm}	0.72 ± 0.03 ^{fghijklm}	0.23 ± 0.00 ^{efghijk}	0.39 ± 0.00 ^{defghijk}	1.89 ± 0.02 ^{efghij}
37	0.28 ± 0.06 ^{p}	0.20 ± 0.05 ^{mno}	0.21 ± 0.04 ^{klmn}	0.63 ± 0.16 ^{o}	0.75 ± 0.06 ^{defghijkl}	0.34 ± 0.03 ^{abcde}	0.35 ± 0.02 ^{fghijklm}	2.05 ± 0.02 ^{cdefghi}
38	0.47 ± 0.07 ^{fghiklmn}	0.25 ± 0.03 ^{jklmno}	0.35 ± 0.06 ^{bcdef}	0.90 ± 0.15 ^{lmno}	0.99 ± 0.05 ^{bcdefg}	0.30 ± 0.01 ^{cde}	0.53 ± 0.02 ^{abcd}	2.27 ± 0.06 ^{abcdefghi}
39	0.38 ± 0.02 ^{lmnop}	0.29 ± 0.03 ^{ghijk}	0.28 ± 0.06 ^{defghijkl}	0.77 ± 0.07 ^{mno}	0.50 ± 0.02 ^{klmn}	0.19 ± 0.00 ^{fghijkl}	0.19 ± 0.01 ^{mn}	1.05 ± 0.02 ^{mnop}
40	0.45 ± 0.02 ^{ghijklmno}	0.33 ± 0.01 ^{cdefg}	0.33 ± 0.01 ^{cdefg}	1.07 ± 0.04 ^{hijklm}	0.88 ± 0.04 ^{bcdefghi}	0.25 ± 0.01 ^{defghi}	0.38 ± 0.02 ^{defghijk}	2.11 ± 0.10 ^{bcdefghi}
41	0.39 ± 0.01 ^{jklmnop}	0.36 ± 0.01 ^{bcde}	0.29 ± 0.01 ^{defghijk}	1.05 ± 0.00 ^{ijklm}	0.88 ± 0.06 ^{cdefghi}	0.29 ± 0.02 ^{cde}	0.39 ± 0.03 ^{defghijk}	2.16 ± 0.13 ^{bcdefghi}

Values are the mean ± standard error (n = 2).

Statistical analysis was performed using one-way analysis of variance (ANOVA) followed by Tukey's multiple comparison test to compare the 41 cultivars for each component, without considering the year factor. Different letters within a column indicate significant differences among cultivars ($p < 0.05$).

Table S6. Carotenoids content identified in 41 wheat cultivars harvested in 2019 and 2020

(μg/100 g whole wheat)

No.	2019				2020			
	Lutein	Zeaxanthin	α-carotene	β-carotene	Lutein	Zeaxanthin	α-carotene	β-carotene
1	123.47 ± 1.71 ^{ac}	20.20 ± 1.62 ^{nop}	ND	ND	79.18 ± 7.12 ^{abcdef}	8.49 ± 0.32 ^{efghijklm}	ND	ND
2	108.37 ± 0.17 ^{defg}	10.18 ± 1.09 ^{p}	ND	ND	65.13 ± 11.32 ^{efghijkl}	8.51 ± 0.94 ^{efghijklm}	ND	ND
3	117.47 ± 0.64 ^{abcd}	25.26 ± 0.36 ^{klomnj}	ND	ND	77.61 ± 11.75 ^{abcefg}	11.25 ± 2.02 ^{bcde}	ND	ND
4	119.55 ± 2.11 ^{abcd}	21.41 ± 1.80 ^{mnop}	ND	ND	98.85 ± 1.13 ^{a}	10.54 ± 0.00 ^{bcdefgh}	ND	ND
5	111.73 ± 0.52 ^{cde}	84.89 ± 3.14 ^{a}	ND	ND	69.43 ± 1.97 ^{abcdefgh}	11.54 ± 0.17 ^{bc}	ND	ND
6	83.76 ± 0.20 ^{jklm}	73.31 ± 1.57 ^{abc}	ND	ND	50.39 ± 2.30 ^{fghijkln}	10.83 ± 0.25 ^{bcdef}	ND	ND
7	132.02 ± 1.46 ^{a}	64.40 ± 1.49 ^{bcd}	ND	ND	69.63 ± 4.35 ^{abcdefgh}	11.60 ± 0.46 ^{bc}	ND	ND
8	126.69 ± 3.38 ^{ab}	74.92 ± 0.86 ^{ab}	ND	ND	91.22 ± 11.38 ^{ab}	12.16 ± 1.55 ^{b}	ND	ND
9	100.96 ± 1.03 ^{efgh}	75.19 ± 3.61 ^{ab}	ND	ND	51.72 ± 2.92 ^{fghijkln}	9.11 ± 0.28 ^{cdefghijklm}	ND	ND
10	93.77 ± 5.13 ^{ghij}	61.42 ± 1.03 ^{d}	ND	ND	54.08 ± 0.50 ^{fghijkln}	10.83 ± 0.89 ^{bcdef}	ND	ND
11	117.55 ± 1.28 ^{abcd}	70.05 ± 6.72 ^{bcd}	ND	ND	84.32 ± 3.12 ^{abcd}	11.53 ± 0.66 ^{bcd}	ND	ND
12	111.09 ± 2.17 ^{cde}	65.79 ± 0.25 ^{bcd}	ND	ND	47.98 ± 1.90 ^{lm}	8.51 ± 0.85 ^{efghijklm}	ND	ND
13	105.21 ± 3.25 ^{defg}	38.13 ± 3.06 ^{fghi}	ND	ND	70.78 ± 1.74 ^{abcdefgh}	8.30 ± 0.07 ^{fghijklm}	ND	ND
14	80.34 ± 4.09 ^{klm}	36.40 ± 0.17 ^{ghij}	ND	ND	56.60 ± 3.71 ^{fghijkl}	8.53 ± 0.13 ^{efghijklm}	ND	ND
15	100.93 ± 8.15 ^{efgh}	46.69 ± 6.04 ^{fg}	ND	ND	83.47 ± 10.33 ^{abcd}	7.59 ± 1.22 ^{ijklm}	ND	ND
16	95.33 ± 5.46 ^{ghi}	38.57 ± 6.82 ^{fghi}	ND	ND	80.24 ± 2.95 ^{abcde}	8.09 ± 0.37 ^{fghijklm}	ND	ND
17	68.39 ± 2.80 ^{n}	37.90 ± 1.90 ^{fghi}	ND	ND	45.01 ± 1.54 ^{lm}	6.01 ± 0.22 ^{m}	ND	ND
18	104.77 ± 3.64 ^{efg}	46.05 ± 0.01 ^{fg}	ND	ND	72.92 ± 3.53 ^{abcdefghi}	7.59 ± 0.00 ^{ijklm}	ND	ND
19	106.86 ± 6.60 ^{defg}	44.74 ± 5.63 ^{fgh}	ND	ND	63.90 ± 7.34 ^{efghijkl}	8.70 ± 1.76 ^{defghijklm}	ND	ND
20	95.59 ± 9.78 ^{fghi}	49.27 ± 2.33 ^{ef}	ND	ND	72.58 ± 4.54 ^{abcdefghi}	9.75 ± 0.66 ^{bcdefghij}	ND	ND
21	95.78 ± 4.47 ^{fghi}	60.36 ± 2.07 ^{ed}	ND	ND	60.07 ± 0.45 ^{efghijkln}	6.52 ± 0.02 ^{lm}	ND	ND
22	98.84 ± 1.83 ^{fgh}	60.82 ± 0.83 ^{ed}	ND	ND	74.12 ± 1.80 ^{abcdefh}	7.77 ± 0.10 ^{hijklm}	ND	ND
23	113.38 ± 0.35 ^{cde}	58.65 ± 0.89 ^{ed}	ND	ND	71.57 ± 4.81 ^{abcdefghi}	8.29 ± 0.67 ^{fghijklm}	ND	ND
24	95.39 ± 5.63 ^{fghi}	60.16 ± 0.21 ^{ed}	ND	ND	43.90 ± 5.23 ^{m}	7.19 ± 0.88 ^{jklm}	ND	ND
25	109.19 ± 0.72 ^{defg}	59.06 ± 1.22 ^{ed}	ND	ND	86.33 ± 5.46 ^{abc}	8.18 ± 0.64 ^{fghijklm}	ND	ND
26	112.04 ± 1.09 ^{cde}	64.76 ± 0.70 ^{bcd}	ND	ND	70.78 ± 0.20 ^{abcdefghi}	8.49 ± 0.20 ^{efghijklm}	ND	ND
27	87.36 ± 3.54 ^{hijk}	62.09 ± 1.22 ^{cd}	ND	ND	64.50 ± 7.79 ^{efghijkl}	8.70 ± 0.06 ^{defghijklm}	ND	ND
28	88.28 ± 0.61 ^{ghijk}	24.05 ± 0.55 ^{klmno}	ND	ND	83.06 ± 1.46 ^{abcd}	10.77 ± 0.11 ^{bcdefg}	ND	ND
29	44.84 ± 1.48 ^{p}	16.10 ± 1.37 ^{op}	ND	ND	74.11 ± 0.32 ^{abcdefh}	9.58 ± 0.13 ^{bcdefghij}	ND	ND
30	45.64 ± 0.12 ^{p}	22.25 ± 1.89 ^{lmno}	ND	ND	64.51 ± 1.53 ^{efghijkl}	10.43 ± 0.14 ^{bcdefgh}	ND	ND
31	78.84 ± 0.85 ^{klm}	33.36 ± 1.71 ^{hijkl}	ND	ND	57.19 ± 4.12 ^{efghijkln}	9.40 ± 0.26 ^{bcdefghijk}	ND	ND
32	61.50 ± 1.74 ^{no}	24.94 ± 4.94 ^{jklmno}	ND	ND	60.32 ± 1.14 ^{efghijkln}	7.99 ± 0.20 ^{ghijklm}	ND	ND
33	75.65 ± 5.90 ^{klmn}	22.27 ± 5.50 ^{lmno}	ND	ND	65.82 ± 0.28 ^{abcdefghi}	6.55 ± 0.03 ^{lm}	ND	ND
34	81.60 ± 1.14 ^{jklm}	25.12 ± 1.16 ^{jklmno}	ND	ND	58.02 ± 4.44 ^{efghijkln}	8.19 ± 0.19 ^{fghijklm}	ND	ND
35	51.33 ± 1.37 ^{op}	42.11 ± 3.60 ^{fghi}	ND	ND	41.00 ± 5.56 ^{n}	11.30 ± 0.07 ^{bcde}	ND	ND
36	69.12 ± 4.28 ^{mn}	33.23 ± 2.69 ^{hijkl}	ND	ND	54.64 ± 5.75 ^{efghijkln}	6.69 ± 0.23 ^{klm}	ND	ND
37	76.51 ± 3.78 ^{klm}	33.00 ± 0.95 ^{ijklm}	ND	ND	57.19 ± 4.12 ^{efghijkln}	7.11 ± 0.40 ^{jklm}	ND	ND
38	117.30 ± 4.16 ^{abcd}	73.85 ± 2.82 ^{ab}	ND	ND	72.12 ± 6.73 ^{abcdefghi}	15.54 ± 0.92 ^{a}	ND	ND
39	40.21 ± 3.07 ^{p}	24.77 ± 0.74 ^{jklmno}	ND	ND	53.52 ± 0.85 ^{fghijkln}	10.75 ± 0.34 ^{bcdefg}	ND	ND
40	99.78 ± 0.56 ^{efgh}	35.01 ± 0.45 ^{ghijk}	ND	ND	69.61 ± 7.73 ^{abcdefgh}	10.33 ± 0.68 ^{bcdefghi}	ND	ND
41	76.50 ± 0.05 ^{klm}	30.88 ± 0.08 ^{ijklmn}	ND	ND	68.00 ± 4.72 ^{abcdefghi}	11.98 ± 0.33 ^{b}	ND	ND

Values are the mean ± standard error ($n = 2$).

Statistical analysis was performed using one-way analysis of variance (ANOVA) followed by Tukey's multiple comparison test to compare the 41 cultivars for each component, without considering the year factor. Different letters within a column indicate significant differences among cultivars ($p < 0.05$).

Table S7. Climate conditions during the growth period in 2019 and 2020

Period	2019			2020		
	Average temperature (℃)	Precipitation (mm)	Solar radiation (h)	Average temperature (℃)	Precipitation (mm)	Solar radiation (h)
1st (Oct.21 ~ Dec.20)	8.4	88.0	442	8.9	78.0	385
2nd (Dec.21 ~ Feb.20)	0.8	52.4	391	3.2	112.4	302
3rd (Feb.21 ~ Mar.20)	6.9	29.1	180	6.9	47.9	208
4th (Mar.21 ~ Apr.20)	10.3	44.1	242	10.6	38.2	248
5th (Apr.21 ~ May.20)	17.0	83.9	221	16.2	75.4	244
6th (May.21 ~ Jun.20)	20.9	52.2	248	22.0	153.8	220
Total growth period (Oct.21 ~ Jun.20)	10.7	349.7	1725	11.3	505.7	1606

Table S8. Two-way ANOVA results for the effects of cultivar, harvest year, and their interaction on phytochemical contents of whole wheat at the composite sample level.

Compound	Source	F value	P value
	Cultivar	26.88	<0.0001
Heptadecylresorcinol (C17:0)	Year	13.45	0.0004
	Cultivar × Year	6.556	<0.0001
	Cultivar	58.49	<0.0001
Nonadecylresorcinol (C19:0)	Year	0.2567	0.6137
	Cultivar × Year	6.876	<0.0001
	Cultivar	34.15	<0.0001
Heneicosylresorcinol (C21:0)	Year	25.00	0.24
	Cultivar × Year	8.107	<0.0001
	Cultivar	24.53	<0.0001
Tricosylresorcinol (C23:0)	Year	887.1	<0.0001
	Cultivar × Year	12.23	<0.0001
	Cultivar	8.741	<0.0001
Campesterol	Year	2348	<0.0001
	Cultivar × Year	10.24	<0.0001
	Cultivar	2.892	<0.0001
Stigmasterol	Year	115.4	<0.0001
	Cultivar × Year	2.257	0.0009
	Cultivar	13.53	<0.0001
β-Sitosterol	Year	3030	<0.0001
	Cultivar × Year	12.36	<0.0001
	Cultivar	31.48	<0.0001
α-Tocopherol	Year	920.0	<0.0001
	Cultivar × Year	15.96	<0.0001
	Cultivar	47.14	<0.0001
α-Tocotrienol	Year	36.46	<0.0001
	Cultivar × Year	38.66	<0.0001
	Cultivar	32.85	<0.0001
β-Tocopherol	Year	346.7	<0.0001
	Cultivar × Year	12.47	<0.0001
	Cultivar	37.49	<0.0001
β-Tocotrienol	Year	668.8	<0.0001
	Cultivar × Year	25.00	<0.0001
	Cultivar	51.82	<0.0001
Lutein	Year	1400	<0.0001
	Cultivar × Year	20.84	<0.0001
	Cultivar	101.5	<0.0001
Zeaxanthin	Year	12818	<0.0001
	Cultivar × Year	92.02	<0.0001

Values represent F-statistics and corresponding P-values from a standard two-way ANOVA with cultivar and harvest year as fixed factors and their interaction included. Because biological replicates were pooled prior to phytochemical analysis, the analysis reflects comparisons among cultivar–year composite samples rather than with-in-cultivar biological variability.

Table S9. Precision for the determination of phytochemicals in whole wheat

Phytochemicals	Compounds	Repeatability[1)]			Reproducibility[2)]		
		Mean	SD	CV(%)	Mean	SD	CV(%)
Alkylresorcinols (mg/100 g)	Heptadecylresorcinol (C17:0)	3.36	0.12	3.70	3.10	0.17	5.42
	Nonadecylresorcinol (C19:0)	22.73	0.42	1.85	22.45	0.50	2.23
	Heneicosylresorcinol (C21:0)	26.11	0.51	1.95	25.91	0.63	2.44
	Tricosylresorcinol (C23:0)	7.76	0.12	1.58	8.13	0.63	7.80
Phytosterols (mg/100 g)	Campesterol	3.45	0.10	2.90	3.53	0.22	6.16
	Stigmasterol	0.38	0.01	3.44	0.37	0.03	7.89
	β-Sitosterol	14.77	0.58	3.89	14.73	1.00	6.76
Vitamin E (mg/100 g)	α-Tocopherol	0.43	0.04	8.58	0.47	0.03	7.25
	α-Tocotrienol	0.20	0.04	17.91	0.20	0.01	6.69
	β-Tocopherol	0.36	0.06	16.89	0.41	0.03	7.63
	β-Tocotrienol	1.19	0.21	17.69	1.27	0.06	4.46
Carotenoids (μg/100 g)	Lutein	57.99	1.09	1.88	59.02	1.99	3.37
	Zeaxanthin	9.44	0.20	2.12	9.73	0.48	4.96

[1)] Repeatability was evaluated using five independent analyses of replicate sample performed on a given day.
[2)] Reproducibility was evaluated using five independent analyses of replicate sample performed on a different day.
SD, standard deviation; CV, coefficient of variation.

Table S10. Accuracy for the determination of phytochemicals in whole wheat

Phytochemicals	Recovery (%)[1)]		
	Mean	SD	CV(%)
Heptadecylresorcinol (C17:0)	96.24	0.71	0.74
β-Sitosterol	92.08	0.56	0.60
α-Tocopherol	90.44	0.70	0.77
Lutein	98.19	4.77	4.85

[1)] Accuracy is a measure of the closeness of the analytical result to the true value determined by analyzing a spiked sample.

Table S11. Major classes of lipid-soluble phytochemicals analyzed in this study, including representative compounds and their reported biological and nutritional functions in wheat grains.

Phytochemical class	Representative compounds analyzed	Major biological / nutritional functions	Relevance in wheat grains
Alkylresorcinols (ARs)	Heptadecylresorcinol (C17:0) Nonadecylresorcinol (C19:0) Heneicosylresorcinol (C21:0) Tricosylresorcinol (C23:0)	Biomarkers of whole-grain wheat and rye intake; antioxidant activity; potential antimicrobial properties	Mainly located in the bran fraction of wheat grains and widely used as indicators of whole-grain consumption
Phytosterols	β-Sitosterol, Campesterol, Stigmasterol	Cholesterol-lowering effects; inhibition of intestinal cholesterol absorption; anti-inflammatory properties	Important lipid-soluble phytochemicals in cereal grains contributing to the nutritional value of whole wheat products
Vitamin E homologues (Tocols)	α-Tocopherol, β-Tocopherol, α-Tocotrienol, β-Tocotrienol	Lipid-soluble antioxidants that protect cellular membranes from oxidative damage; contribute to human vitamin E intake	Present in wheat germ and bran fractions and contribute to the antioxidant capacity of wheat-based foods
Carotenoids	Lutein, Zeaxanthin	Antioxidant activity; lutein and zeaxanthin support eye health; contribute to pigmentation and nutritional quality	Responsible for yellow pigmentation in wheat grains and important for nutritional quality of wheat products

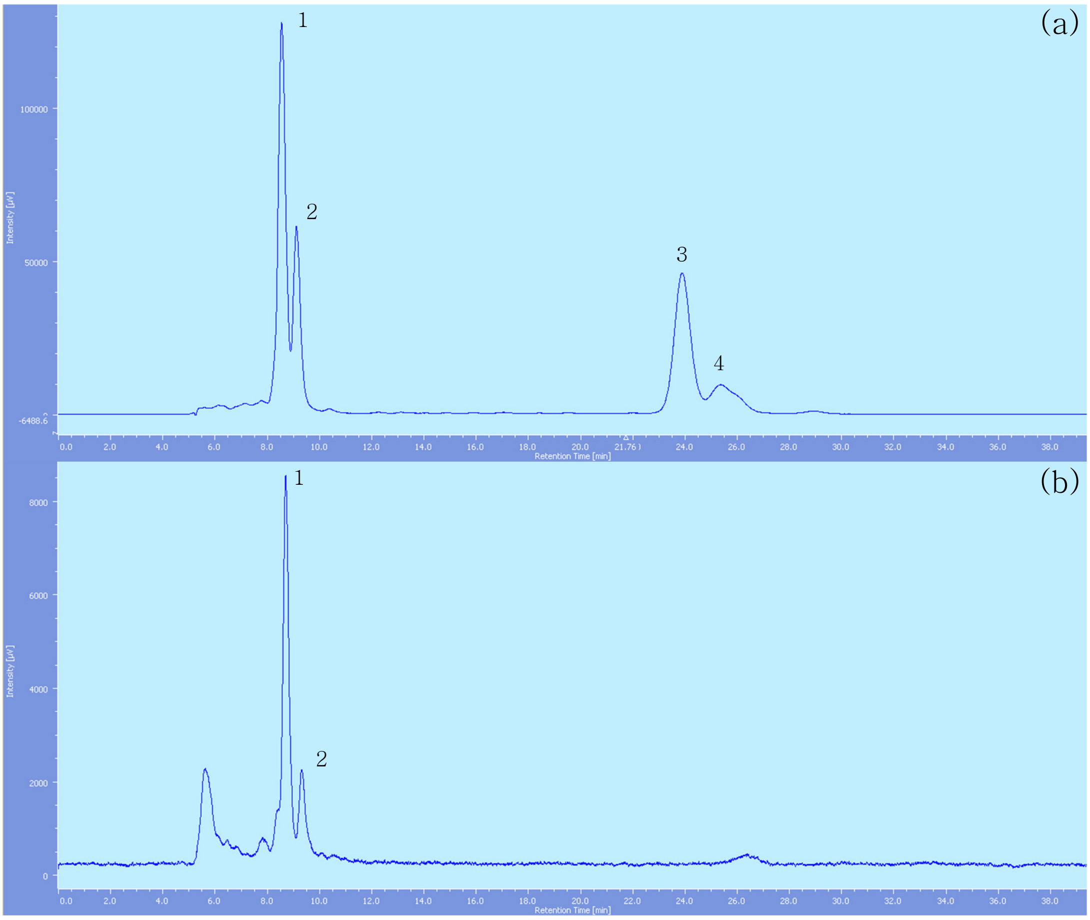

Figure S1. Representative HPLC chromatograms used for the identification of carotenoids in whole wheat samples. (a) carotenoid standards; (b) chromatogram of the wheat cultivar Saekeumkang showing detected carotenoids (1, lutein; 2, zeaxanthin; 3, α-carotene; 4, β-carotene).

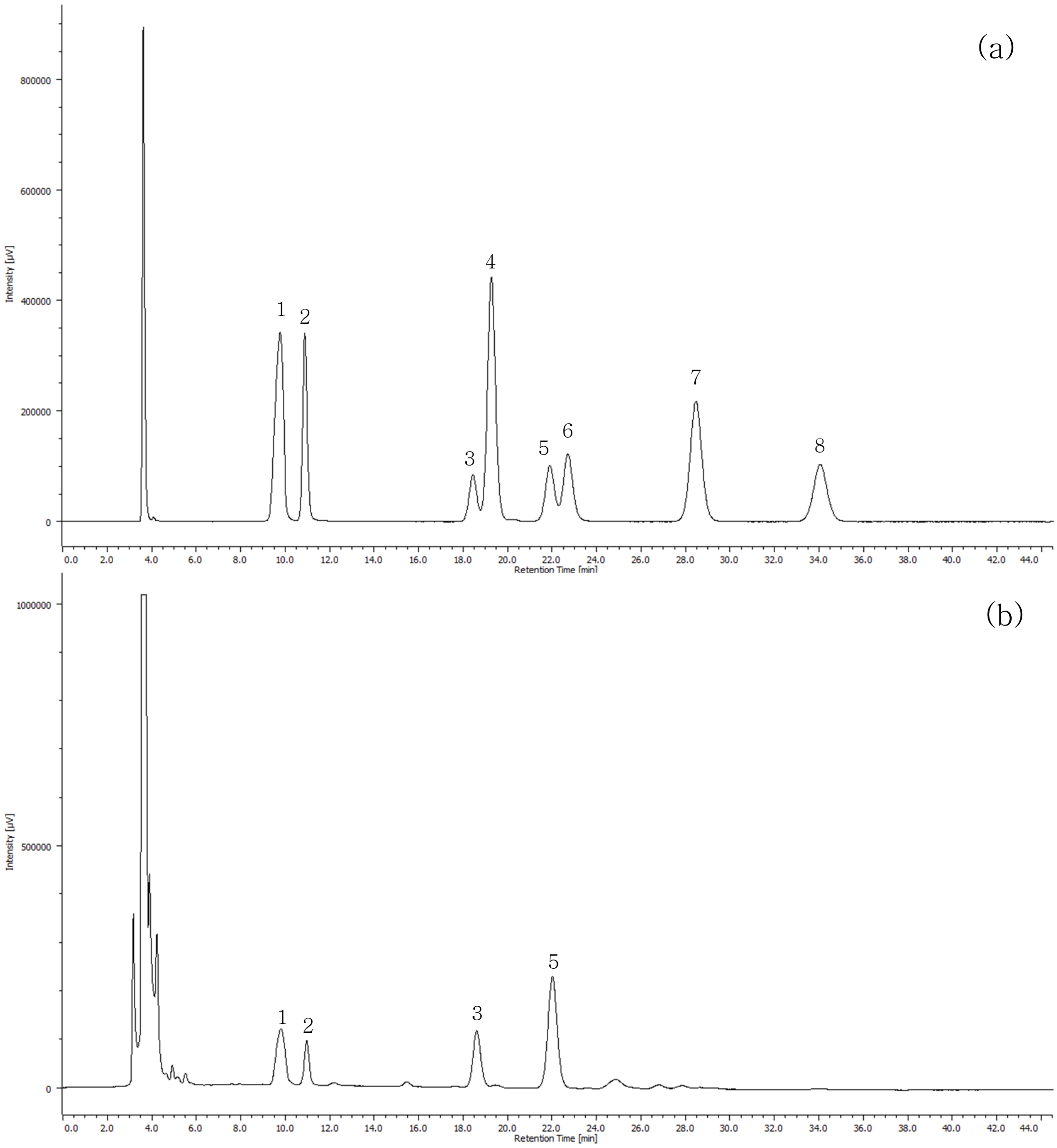

Figure S2. Representative HPLC chromatograms used for the identification of vitamin E compounds in wheat samples. (a) vitamin E standards; (b) chromatogram of the wheat cultivar Saekeumkang showing detected compounds (1, α-tocopherol; 2, α-tocotrienol; 3, β-tocopherol; 4, γ-tocopherol; 5, β-tocotrienol; 6, γ-tocotrienol; 7, δ-tocopherol; 8, δ-tocotrienol).

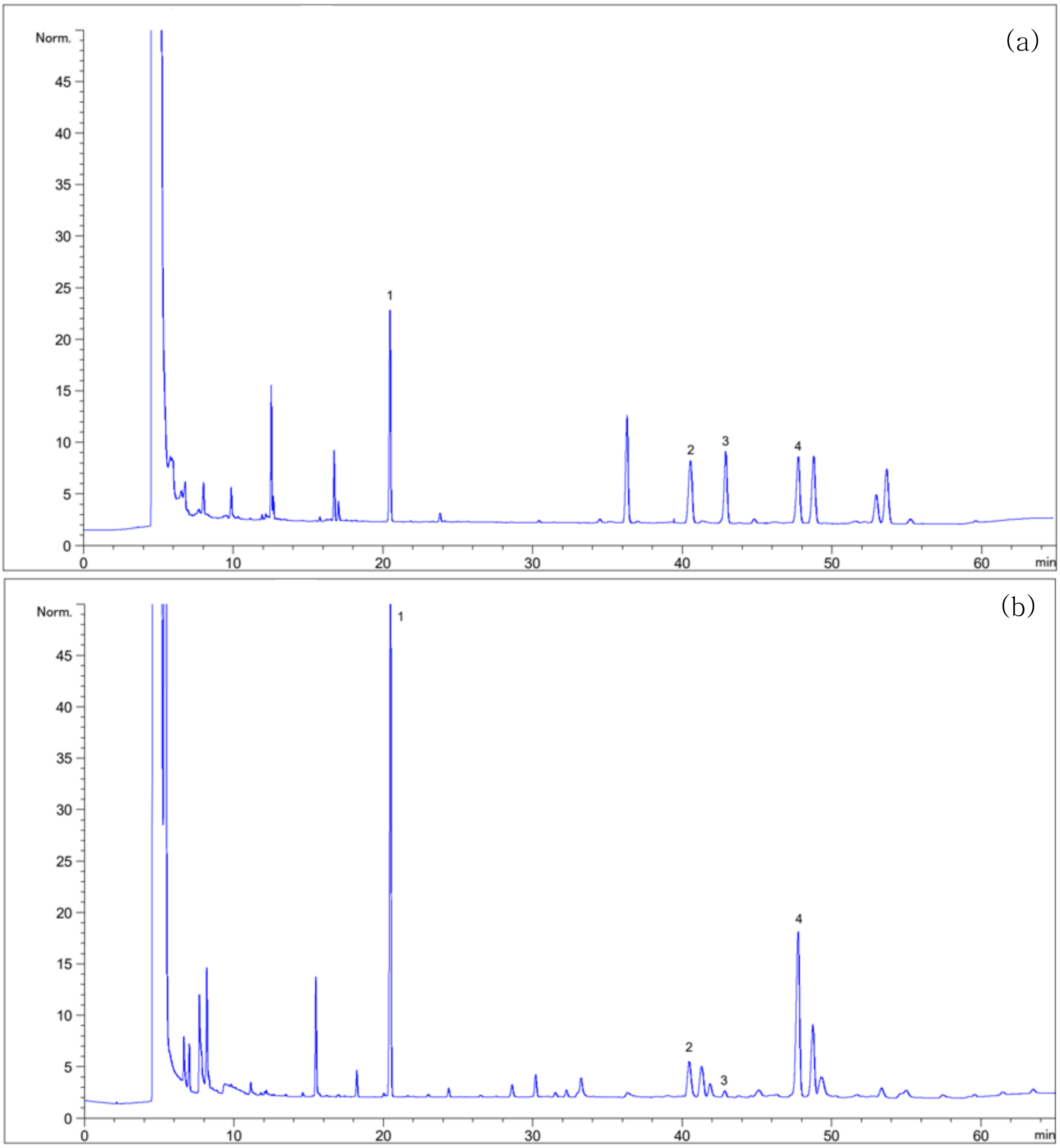

Figure S3. Representative GC chromatograms used for the identification of phytosterols in wheat samples. (a) phytosterol standards; (b) chromatogram of the wheat cultivar Saekeumkang showing detected compounds (1, 5α-cholestane, internal standard; 2, campesterol; 3, stigmasterol; 4, β-sitosterol).

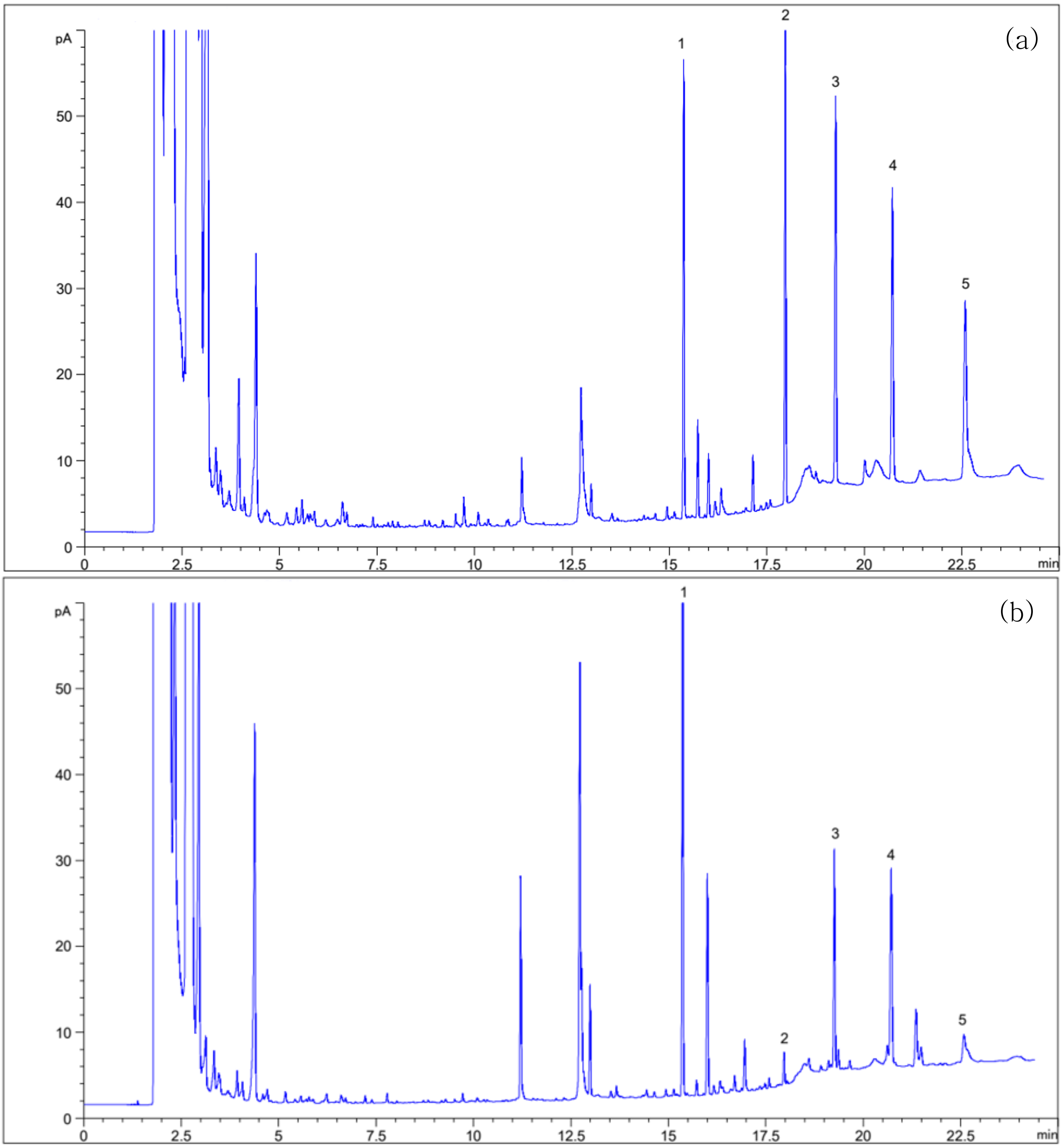

Figure S4. Representative GC chromatograms used for the identification of alkylresorcinols in wheat samples. (a) alkylresorcinol standards; (b) chromatogram of the wheat cultivar Saekeumkang showing detected compounds (1, methyl behenate, internal standard; 2, 5-n-heptadecylresorcinol; 3, 5-n-nonadecylresorcinol; 4, 5-n-heneicosylresorcinol; 5, 5-n-tricosylresorcinol).
